# What to Choose for Estimating Leaf Water Status—Spectral Reflectance or In vivo Chlorophyll Fluorescence?

**DOI:** 10.34133/plantphenomics.0243

**Published:** 2024-08-29

**Authors:** Martina Špundová, Zuzana Kučerová, Vladimíra Nožková, Monika Opatíková, Lucie Procházková, Pavel Klimeš, Jan Nauš

**Affiliations:** ^1^ Department of Biophysics, Faculty of Science, Palacký University, Šlechtitelů 27, Olomouc 783 71, Czech Republic.; ^2^ Department of Chemical Biology, Faculty of Science, Palacký University, Šlechtitelů 27, Olomouc 783 71, Czech Republic.; ^3^ Czech Advanced Technology and Research Institute (CATRIN), Palacký University Olomouc, Šlechtitelů 27, Olomouc, 783 71, Czech Republic.

## Abstract

In the context of global climate change and the increasing need to study plant response to drought, there is a demand for easily, rapidly, and remotely measurable parameters that sensitively reflect leaf water status. Parameters with this potential include those derived from leaf spectral reflectance (R) and chlorophyll fluorescence. As each of these methods probes completely different leaf characteristics, their sensitivity to water loss may differ in different plant species and/or under different circumstances, making it difficult to choose the most appropriate method for estimating water status in a given situation. Here, we present a simple comparative analysis to facilitate this choice for leaf-level measurements. Using desiccation of tobacco (*Nicotiana tabacum* L. cv. Samsun) and barley (*Hordeum vulgare* L. cv. Bojos) leaves as a model case, we measured parameters of spectral R and chlorophyll fluorescence and then evaluated and compared their applicability by means of introduced coefficients (coefficient of reliability, sensitivity, and inaccuracy). This comparison showed that, in our case, chlorophyll fluorescence was more reliable and universal than spectral R. Nevertheless, it is most appropriate to use both methods simultaneously, as the specific ranking of their parameters according to the coefficient of reliability may indicate a specific scenario of changes in desiccating leaves.

## Introduction

One of the major problems associated with global climate change is drought, which is expected to increase in frequency and severity in many regions of the world. Drought has a substantial impact on plant growth and productivity, so investigating plant responses and resilience to drought conditions is imperative. In parallel, attention is being paid to methods for monitoring plant water status, often in the context of phenotyping, remote sensing, or irrigation scheduling [1–5]. For large-scale studies, methods that allow nondestructive, high-throughput assessment of plant water status are highly sought after.

One method with such potential is the measurement of spectral leaf reflectance (R) at the leaf, plant, or canopy level. Water, which is the most abundant substance in leaf tissue, significantly affects the optical properties of leaves, including R. A direct and indirect effect of water content on leaf R can be distinguished [6,7]. The direct effect is caused by absorption in the shortwave infrared (SWIR) region (1,400 to 3,000 nm), which is mainly manifested by pronounced absorption bands at 1,450, 1,940 and 2,500 nm. As leaves dehydrate, R in these bands increases [6,8,9]. Water also absorbs radiation in the near-infrared (NIR) region (760 to 1,400 nm), but the absorption in this region is relatively low [10]. However, an absorption band at ~970 nm has been reported to be sensitive to leaf water content [6,11–13].

The indirect effect of water on leaf R is related to other leaf properties that change with the leaf dehydration. These include leaf structural characteristics and pigment content [particularly chlorophyll (Chl)] [7,14–16]. One of the parameters primarily reflecting Chl content, but also commonly used as a “plant water estimator” in remote sensing, is a normalized difference vegetation index (NDVI) [3]. This parameter has been reported to be closely related to changes in leaf water status [9,17].

While Chl content only affects R in the visible (VIS) region (390 to 760 nm), leaf structure affects R in all VIS, NIR, and SWIR regions [7,14,15]. However, Chl content and leaf structure are also dependent on plant species, genotype, age, nutrient status, and growth conditions [16,18,19], making the use of related parameters to estimate leaf water status problematic. Currently, the hyperspectral measurement of R is recommended to increase the chance of a sensitive response of R to changing leaf water status and to obtain more complex information on changes in leaf optical properties [11–15,20,21].

Leaf water status also affects physiological processes, including photosynthesis. The standard method for assessing photosynthetic function is the measurement of Chl fluorescence in vivo, which is relatively rapid, is nondestructive, and, in certain configurations, can be successfully used for remote sensing [2,4,22]. There are a number of Chl fluorescence parameters that are more or less sensitive to changes in the leaf water status [4,23–29]. Measurement of Chl fluorescence is often used not only to monitor leaf water status [2,22,25] but also to study effects of water deficit on plants and has contributed to understanding the mechanism of action of leaf water shortage on photosynthesis [23,24]. On the other hand, not all Chl fluorescence measurements are suitable for remote sensing. Standard procedures require pre-darkening of the samples before measurement, and for a sensitive response of most parameters, measurement times in the order of minutes are required. However, more modern Chl fluorescence methods overcome these limitations and, for example, sun-induced Chl fluorescence (SIF) has been successfully used in remote sensing to monitor plant response to drought [30,31].

Spectral R and Chl fluorescence probe different leaf characteristics, which has already been used to improve the interpretation of drought-induced changes in SIF by simultaneously measuring R in remote sensing studies [31–33]. Sensitivity of R and Chl fluorescence to leaf water status may differ in different plant types and species and/or under different circumstances (e.g., plant age, drought conditions, and the influence of other stress factors), making it difficult to choose the more sensitive method for estimating water status in a given situation. Although many indices derived from spectral R have been found to correlate with leaf water content [3,33], some studies have reported the sensitivity of R to leaf water status to be low [6,7,16,21,34,35]. A similar situation exists in the case of Chl fluorescence, where different sensitivities of its parameters have been reported in different cases [4,23–29]. If a choice has to be made, there is still insufficient information when it is more appropriate to use spectral R or Chl fluorescence to estimate leaf water status.

To date, little attention has been paid to comparing spectral R and Chl fluorescence changes during rapid desiccation of higher plants’ leaves. This question has been addressed almost exclusively in poikilohydric mosses and lichens, which are characterized by a unique desiccation tolerance [36,37]. This is understandable because under natural conditions, desiccation of higher plant leaves rarely occurs. However, due to the high rate of water loss during leaf desiccation, when acclimation processes are minimized, changes in spectral R and Chl fluorescence parameters more directly reflect water loss-induced structural and photosynthetic changes in the leaf. Comparing them could help to identify leaf characteristics that may play a role in plant drought tolerance or resistance. The information gained from such measurements can be subsequently used in remote sensing technologies and models to improve the prediction of plant stress response to drought, for example, in relation to crop yield.

In our study, we therefore compared the sensitivity of R-derived and Chl fluorescence parameters to the decrease in relative water content (RWC) during desiccation of detached leaves. For this comparison, taxonomically distinct plants tobacco (dicotyledon with bifacial leaves) and barley (monocotyledon with monofacial leaves) were chosen as representatives of important crops. We analyzed changes in spectral R in the VIS, NIR, and SWIR regions measured by different methods and instruments, and in Chl fluorescence parameters reflecting function of photosystem II (PSII) photochemistry. In order to compare the parameters in terms of their applicability for assessing leaf water status, specific coefficients [the coefficient of reliability (*CR*), the coefficient of sensitivity (*CS*), and the coefficient of inaccuracy (*CI*)] have been introduced.

## Materials and Methods

### Plant material and experimental design

**Fig. 1.**
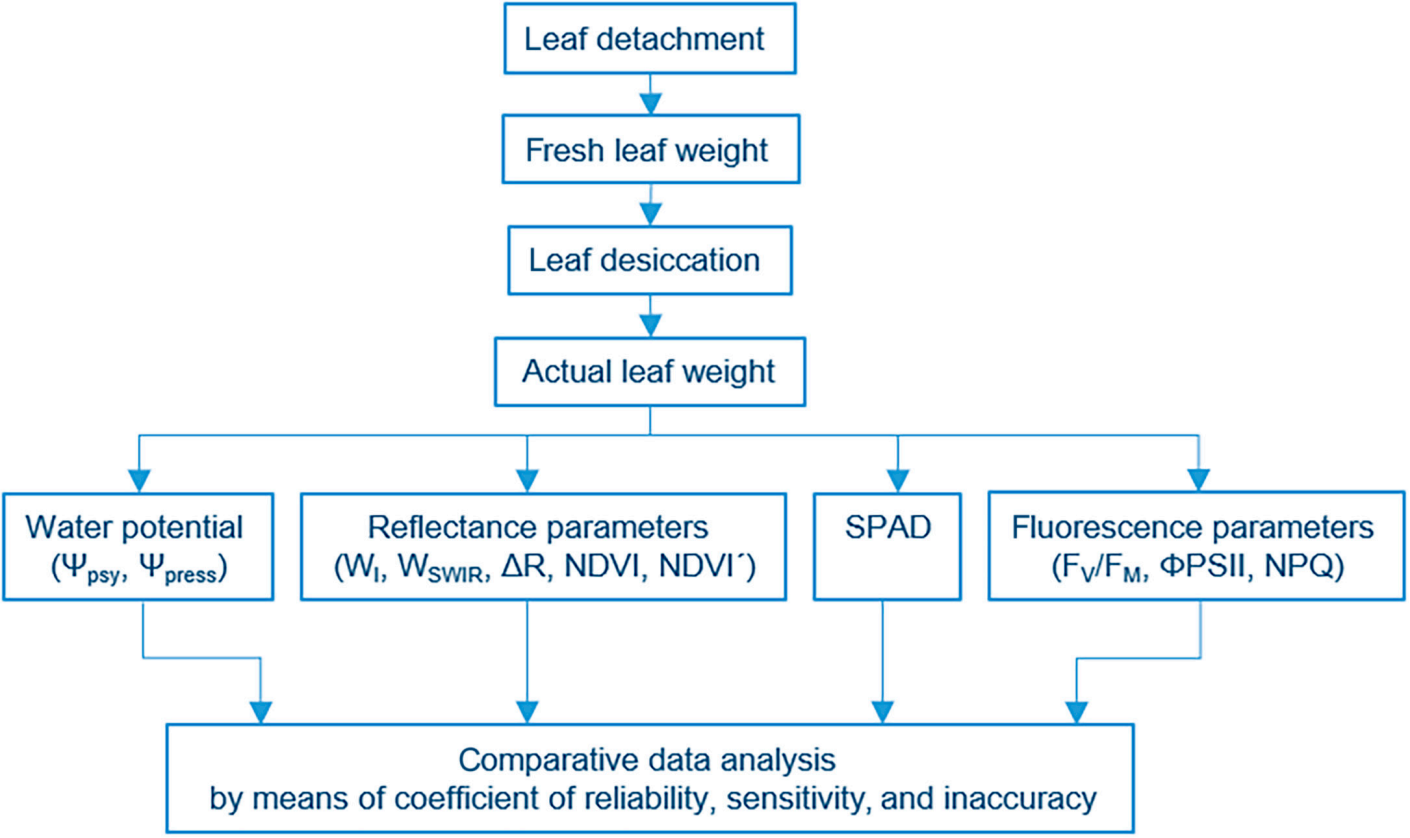
Workflow and parameters used for comparative analysis.

Tobacco (*Nicotiana tabacum* L. cv. Samsun) and barley (*Hordeum vulgare* L. cv. Bojos) plants were grown in a growth chamber under 8 h:16 h, dark:light (white light of 100 μmol photons m^−2^ s^−1^; fluorescent tubes), 60% relative air humidity, and temperature 21 °C. The tobacco plants were grown in a soil substrate (Potgrond H, Klasmann-Deilmann, Geeste, Germany) and watered regularly, and the barley plants were grown in perlite soaked with Knop solution. Tobacco leaves (of similar size and Chl content) were excised from ~2-month-old plants in the vegetative growth phase. Whole leaves including petioles or circular leaf segments (diameter 14 mm) were left to desiccate. For barley, leaf segments were detached 5 cm from the apex of the first mature leaves (10 to 12 days after seed sowing). Immediately after detachment, the fresh weight (m_f_) of each leaf sample was determined and then the samples were placed on plates with dry filter paper and desiccated in the growth chamber under the same conditions as the plants had been grown. After different periods of desiccation, the actual weight (m_a_) of each leaf sample was determined, selected parameters were measured, and comparative analysis was carried out (Fig. 1).

The primary water status parameter used was RWC estimated from a leaf mass decrease (LMD) defined as LMD = (m_f_ − m_a_) ∙ 100/m_f_ (%). In the case of longer measuring procedures (diffusive R and Chl fluorescence parameters in the light-adapted state), m_a_ was determined as the average of the leaf weight before and after the measurement. In a separate experiment, we determined a correlation between LMD and RWC. RWC was determined as RWC = (m_a_ − m_d_) ∙ 100/(m_t_ − m_d_) (%), where m_t_ is a weight of fully turgescent leaf and m_d_ is a dry weight. As there was a strong linear correlation between LMD and RWC (*R*^2^ = 0.994), in further experiments, RWC was calculated as follows: for tobacco, RWC = −1.1085 ∙ LMD + 100.37; for barley, RWC = −1.0986 ∙ LMD + 98.221. In some cases, an equivalent water thickness (EWT; the leaf water content per unit leaf area) was estimated as EWT = (m_a_ − m_d_)/A_a_ (g∙cm^−2^), where m_a_ is the actual leaf weight, m_d_ is the dry weight, and A_a_ is an actual area of a leaf sample. In most cases, a leaf with a given RWC was used for measurement by only one of the methods described below, except for SPAD (relative Chl content measured by a SPAD-502 chlorophyll meter) and NDVI′_D_ measurements (see below), which were made on the same leaves and repeatedly during their desiccation.

### Leaf water potential

Leaf water potential was measured psychrometrically (ψ_psy_) and using a pressure chamber (ψ_press_). ψ_psy_ was measured using a C-52 psychrometric chamber and an HR-33T Dew Point Microvoltmeter (Wescor Inc., Logan, Utah, USA) after 2 h of equilibration of a sample in the chamber. ψ_press_ was measured by a pressure chamber PMS 600 (PMS Instrument Company, Albany, Oregon, USA) using compressed nitrogen gas. During the pressurization of the chamber (by a rate of 0.02 to 0.03 MPa s^−1^), an image of the cutting surface of the petiole/leaf blade was taken using an industrial microscope and a simultaneous image of the pressure gauge was taken using a web camera. These recordings were then used for a more accurate assessment of ψ_press_.

### Measurement of diffusive R and derived parameters in VIS/NIR (400 to 1,100 nm)

Measurement of diffusive R was realized using a portable spectroradiometer LI-1800 with an integrating sphere LI-1800-12 (LI-COR Inc., Lincoln, Nebraska, USA). The samples were placed and fixed in a double mask made from a filter paper with circular opening of 15 mm diameter. This arrangement allowed to measure the diffusive R from both adaxial (R_D_) and abaxial (R_B_) leaf side. In case of tobacco, the measured area was selected in the central part of the leaf blade near the central vein, excluding the vein. Several segments of barley leaf blades were placed in the mask to fully cover the opening without mutual overlapping. The illuminator equipped with halogen bulb illuminated a standard and sample by continuous light in the range of 400 to 1,100 nm. The spectral slit width of the monochromator was 6 nm. A water index (WI) was estimated according to [11] as WI = R_900_/R_970_, where R_900_ and R_970_ mean R at 900 and 970 nm, respectively.

**Fig. 2.**
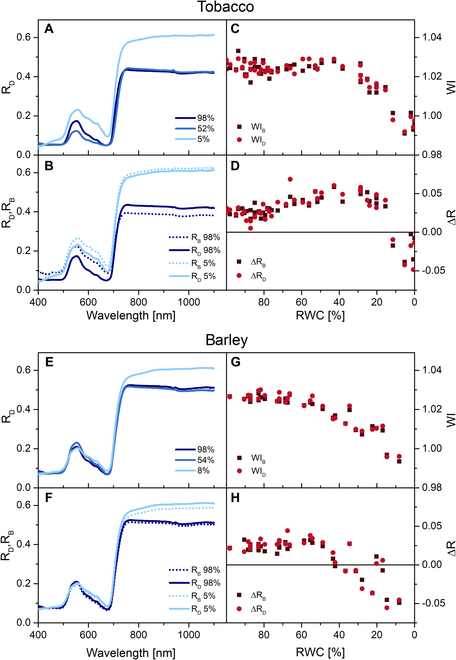
Representative spectra of diffusive R measured on the adaxial side (R_D_) of fresh (RWC = 98%), partially desiccated (RWC = 52% or 54%), and severely desiccated leaves (RWC = 5%) of tobacco (A) and barley (E). Comparison of R_D_ and R_B_ (R from the abaxial leaf side) in fresh and severely desiccated leaves of tobacco (B) and barley (F). WI (WI = R_900_/R_970_) and relative decrease of R in the 800- to 1,100-nm region (ΔR) estimated from R_D_ (indexed by “D”) and R_B_ (indexed by “B”) in desiccating leaves of tobacco (C and D) and barley (G and H). Each point in (C), (D), (G), and (H) represents a single leaf sample. RWC was estimated from the average of the sample weight before and after the measurement.

The spectrum of R in the interval 800 to 1,100 nm was rather linear having decreasing or increasing character (see Fig. 2). This behavior was characterized by a quantity ΔR:

$$∆R=\frac{R_{800}-R_{1100}}{R_{800}},$$ (1)

where R_800_ and R_1100_ mean R at 800 and 1,100 nm, respectively.

### Measurement of R in SWIR (1,000 to 1,700 nm)

R between 1,000 and 1,700 nm was measured in a hyperspectral imaging station (PSI s.r.o., Drásov, Czech Republic) from adaxial side of leaf samples. The station consisted of a SWIR hyperspectral camera (PSI s.r.o., Drásov, Czech Republic), a halogen tube (Clear Infrared Halogen Tube, Nantong Huayu Electrical Lighting Co. Ltd., JianKang RD, Sijia Town, Haimen City, Jiangsu, China), and an RGB (red, green, and blue) camera (GigE μEye UI-5580CP-C-HQ). The camera faced the sample perpendicularly on the center of measured tray with the distance of about 380 mm and was calibrated automatically in respect of white and dark calibration before every measurement. Normalized values of leaf R were derived from complementary energy flux measurements of white reference standard (polytetrafluorethylene, 450 × 50 × 10 mm; TRIBON s.r.o., Brno, Czech Republic) and dark current, where each pixel was calibrated per se (space calibration).

The halogen tube (400 × 100 mm, 600 W) illuminating the scene under the SWIR camera was supplemented with a parabolic mirror focusing the light toward the sample. The light from the halogen tube was focused on the sample within the camera view at a distance of ~380 mm and an angle of 8.7°. The RGB camera was integrated with a 4.92-megapixel complementary metal-oxide-semiconductor sensor (MT9P006STC) for precise plant feature recognition and mask co-registration with SWIR camera readout. About 13 mm in size, the sensor delivered a resolution of 2,560 × 1,920 pixels as well as rolling and global start shutter features. Additional light-emitting diodes (white light, ~400 μmol photons m^−2^ s^−1^) provided uniform illumination for the RGB camera across scanning area. PlantScreen Data Analyser software (PSI s.r.o., Drásov, Czech Republic) was used to pre-process the hyperspectral data. We have defined a parameter WI_SWIR_ as WI_SWIR_ = R_1000_/R_1450_, where R_1000_ and R_1450_ mean R at 1,000 and 1,450 nm. The software was used to calculate a mean WI_SWIR_ value over the whole area of the measured leaf samples. Two-dimensional images of WI_SWIR_ in the leaf areas were extracted from the hyperspectral data by image processing.

### Estimation of NDVI

NDVI was estimated from the measurement of diffusive R on both the abaxial side (NDVI_B_) and adaxial side (NDVI_D_) of the leaf using the LI-1800 spectroradiometer (LI-COR Inc., Lincoln, Nebraska, USA) as NDVI = (R_780_ − R_630_)/(R_780_ + R_630_), where R_780_ and R_630_ are R at 780 and 630 nm. In some cases, NDVI was also estimated from directional R measured with a PolyPen RP 400 (PSI s.r.o., Drásov, Czech Republic) on the adaxial leaf side (NDVI′_D_).

### Measurement of SPAD value

A SPAD value was measured by a SPAD-502 chlorophyll meter (Konica Minolta Sensing Inc., Osaka, Japan) from the adaxial leaf side. The SPAD value is estimated from leaf transmittance at 650 and 940 nm [38].

### Chl fluorescence measurement

Chl fluorescence parameters were measured using a FluorCam imaging system FC 800-O (PSI s.r.o., Drásov, Czech Republic) on adaxial side of pre-darkened (30 min) leaf samples at room temperature. First, a maximum quantum yield of PSII photochemistry [F_V_/F_M_ = (F_M_ − F_0_)/F_M_] was determined, where F_M_ and F_0_ are the maximal and minimal fluorescence of dark-adapted sample, respectively. F_0_ was measured by applying several microsecond-long measuring flashes (red light, 0.1 μmol photons m^−2^ s^−1^), and F_M_ was measured using a saturating pulse (blue light, length 800 ms, 3,500 μmol photons m^−2^ s^−1^). After 2 min of dark relaxation, the measured leaf sample was exposed for 10 min to actinic light (red light, 200 μmol photons m^−2^ s^−1^) and a series of saturating pulses (blue light, 3,500 μmol photons m^−2^ s^−1^) was used to measure the maximal fluorescence in the light-adapted sample (F_M_′) during Chl fluorescence induction. The following parameters were estimated: an effective quantum yield of PSII photochemistry in the light-adapted (steady) state ΦPSII_st_ = (F_M_′_st_ − F_st_)/F_M_′_st_), a nonphotochemical quenching of Chl fluorescence (NPQ) after 1 min of exposure to the actinic light [NPQ_1_ = (F_M_ − F_M_′_1_)/F_M_′_1_], and NPQ at steady state [NPQ_st_ = (F_M_ − F_M_′_st_)/F_M_′_st_]. F_M_′_st_ and F_M_′_1_ are the maximal fluorescence at steady state (i.e., after 10 min of the actinic light) and after 1 min of the actinic light, and F_st_ is fluorescence at steady state. Mean values of parameters over the whole area of the measured leaf samples were calculated and presented.

### Introduction of a coefficient of reliability (*CR*), coefficient of sensitivity (*CS*), and coefficient of inaccuracy (*CI*)

In order to compare different methods or parameters used to monitor changes in leaf properties related to the level of hydration, we introduced a coefficient of reliability (*CR*). RWC has been chosen as the basic changing quantity. For our quantifications, we select an RWC interval of 100 to 50% because a decrease in leaf RWC below ~50% is usually associated with severe leaf damage that is lethal [39]. To assess the suitability of the methods, it is therefore more meaningful to compare them during milder leaf desiccation, i.e., before RWC drops to 50%. The dependence of the parameter on RWC in the interval between 100% and 50% was plotted and fitted by a linear dependence *y_l_(x)* (obtained by a method of least squares):

$$y_{l}\left(x\right)=a+\mathrm{bx}$$ (2)

The coefficient *b* (the slope of the linear interpolation) represents the sensitivity of the parameter (method) to the decrease in RWC. A deviation of the measured value *y(x_i_)* in the point *x_i_* from the linear approximation is:

$$∆y\left(x_{i}\right)=y\left(x_{i}\right)-y_{l}\left(x_{i}\right)$$ (3)

We can now choose (arbitrarily) an RWC interval of interest, Δs, at which a significant change in the measured quantity should be detected. We suggest a value of Δs = 0.1 (10%) for the calculations.

The other side of the measurement process is the accuracy of the instrument, the variability of the leaf samples, and the experience of the investigator. Together, these factors determine the statistical scatter of the measured values. A mean absolute value of the deviation from the linear approximation in the selected RWC interval (*n* points) is:

$$∆\bar{y}=\frac{1}{n}\sum_{i=1}^{n}\left|y\left(x_{i}\right)-y_{l}\left(x_{i}\right)\right|$$ (4)

The quantity *ȳ* is the mean value of *y_i_*:

$$\bar{y}=\frac{1}{n}\sum_{i=1}^{n}y_{i}$$ (5)

The coefficient of reliability *CR* can be defined using the quantities introduced above as:

$$\mathrm{CR}=\frac{b.∆s}{∆\bar{y}}$$ (6)

Considering *b* and Δ*ȳ* separately, we can describe a basic sensitivity and accuracy (or inaccuracy) of the obtained data. We will therefore introduce 2 additional coefficients:

the coefficient of sensitivity (*CS*), defined as:

$$\mathrm{CS}=100\;\frac{b}{\bar{y}}\;$$ (7)

and the coefficient of inaccuracy (*CI*), defined as:

$$\mathrm{CI}=100\;\frac{∆\bar{y}}{\bar{y}}$$ (8)

while multiplication by 100 was used to obtain the highest values of the coefficients in the order of units (*CS*) or tens (*CI*).

The next step is the selection of a reliability threshold value. For example, a critical value of *CR* may be 1. For *CR* > 1, the linear increase in the interval is greater than the mean of the absolute deviation and the dependence is clear and may be statistically significant. A more appropriate value of the critical *CR* can be determined by using statistical methods and requiring a certain level of significance (e.g., the α parameter). Although the concept of the critical *CR* value may be questionable, the quantity *CR* allows us to rank the methods according to this coefficient and to compare them with regard to reliability. Taking into account the actual values obtained for *CR* across all measured parameters, *CR* = 0.4 was chosen as the critical value.

## Results

### Changes in spectral R and R-derived parameters

There were minimal changes in the spectra of diffusive R in VIS and NIR in desiccating tobacco leaves with RWC up to 50% (Fig. 2A) (for a rate of their RWC decrease, see Fig. S1). The R band at ~550 nm decreased, while NIR-R was almost unchanged, including less obvious trough at 970 nm. Accordingly, WI (WI = R_900_/R_970_) from both abaxial (WI_B_) and adaxial (WI_D_) leaf side did not decrease in this RWC range (Fig. 2C). Only severely dehydrated leaves showed a pronounced increase in R in both VIS and NIR region in tobacco (Fig. 2A). A decrease in WI_B_ and WI_D_ started at RWC of about 40% (Fig. 2C). Although convincing, the reduction in WIs was only about 3% for nearly dry leaves.

In the fresh tobacco leaves, differences between R measured from the adaxial (R_D_) and abaxial (R_B_) leaf side typical for bifacial leaves were found (see Fig. S2A showing the internal structure of a fresh tobacco leaf). In the leaves with low RWC, the difference between R_D_ and R_B_ was attenuated (Fig. 2B), which is typical for desiccating leaves [7].

The parameter ΔR, which reflects a slope of R spectrum in the interval from 800 to 1,000 nm, was further evaluated. During desiccation, ΔR in tobacco leaves initially slightly increased, which indicates deepening of the R decline behind 800 nm. In leaves with RWC below 20%, there was a change in the slope of the spectrum in the 800- to 1,100-nm region from /decreasing to increasing, as quantified by a change in ΔR values from positive to negative (Fig. 2D). This qualitative change could correspond to a strong irreversible damage to leaf internal structure, probably associated with the predominance of scattering effects over reflection [40,41].

The same measurement was carried out on monofacial barley leaves (see Fig. S2B for the barley leaf structure). In this case, R in VIS almost did not change and the increase in NIR-R in strongly desiccated leaves was less than in tobacco (Fig. 2E). However, WI_B_ and WI_D_ started to decline already at ~60% RWC (Fig. 2G). Similarly, ΔR changed from positive to negative at higher RWC (about 40%) than in tobacco (compare Fig. 2D and 2H).

**Fig. 3.**
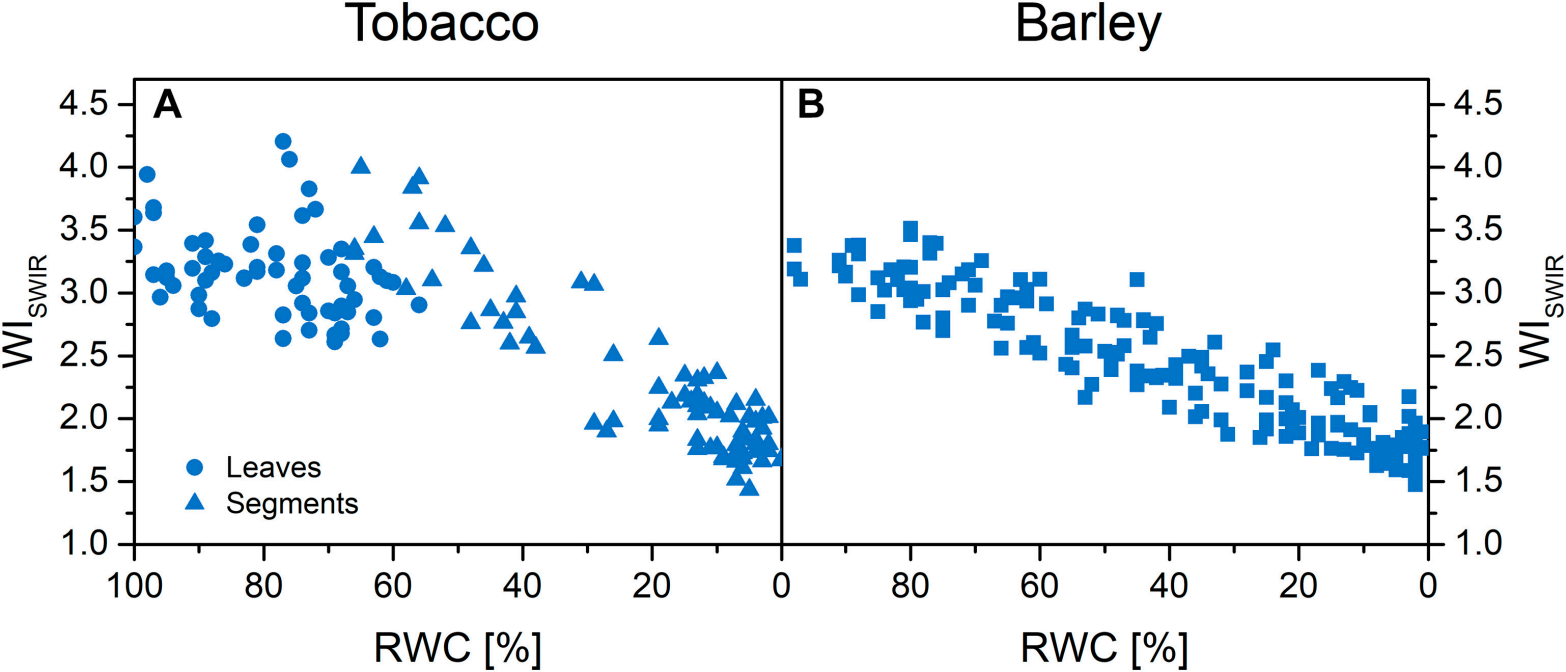
WI (WI_SWIR_ = R_1000_/R_1450_) estimated from measurement of directional R from adaxial side of desiccating leaf samples of tobacco (A) and barley (B).

As WIs derived from NIR-R were not very sensitive to the RWC decrease, we used a SWIR camera to measure R_D_ in the 1,000- to 1,700-nm range, which includes a more pronounced water absorption band at 1,450 nm, and evaluated WI_SWIR_ = R_1000_/R_1450_. WI_SWIR_ was about 3 times higher than WIs and, in nearly dry leaves, was only about half that of fresh leaves (Fig. 3). However, WI_SWIR_ sensitivity to the decreasing RWC remained quite low mainly in tobacco, where it started to decrease only at about 50% RWC, regardless of whether whole leaves or leaf discs were desiccated (Fig. 3A). In barley, the WI_SWIR_ decrease already started at about 80% RWC (Fig. 3B). In the case of tobacco leaves, no significant heterogeneity of WI_SWIR_ was observed through the leaf area; in the case of more desiccated leaf segments, different WI_SWIR_ values were visible at their edges compared to the central part, which could be caused by the curling of the edges (Fig. S3). Similarly, WI_SWIR_ was slightly different at the edges of barley leaves.

**Fig. 4.**
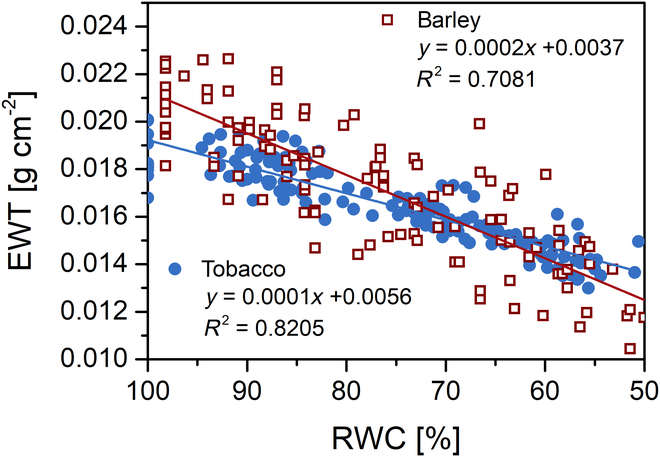
Equivalent water thickness (EWT) during desiccation of tobacco and barley leaf samples within the RWC interval 100% to 50%.

Thus, our expectation based on the published results [6,11–13,42] that spectral R and R-derived WIs would sensitively reflect leaf water status was not fulfilled, especially in the case of tobacco. One of the reasons for low R sensitivity may be the fact that leaves shrink during desiccation. When RWC decreased to 50%, the tobacco leaves decreased their area by ~30%, while barley leaves by ~8% (Fig. S4A). EWT can be used to describe the change in water content relative to leaf area (or the water concentration on area basis), as it has a direct physical relationship with leaf absorption [43]. We found that during the decrease in RWC from 100% to 50%, EWT decreased twice as slowly in tobacco as in barley (Fig. 4). We assume that the tobacco leaves, in contrast to barley, also shrank in thickness, as indicated by a decrease in the R/transmittance ratio at 850 nm (about 13% in tobacco, no decrease in barley; data not shown), which can be used as a predictor of leaf thickness [19]. Thus, the more pronounced shrinkage of desiccating tobacco leaves associated with the slower decrease in EWT could be one of reasons for the low sensitivity of WIs and WI_SWIR_.

### Changes in NDVI and SPAD

**Fig. 5.**
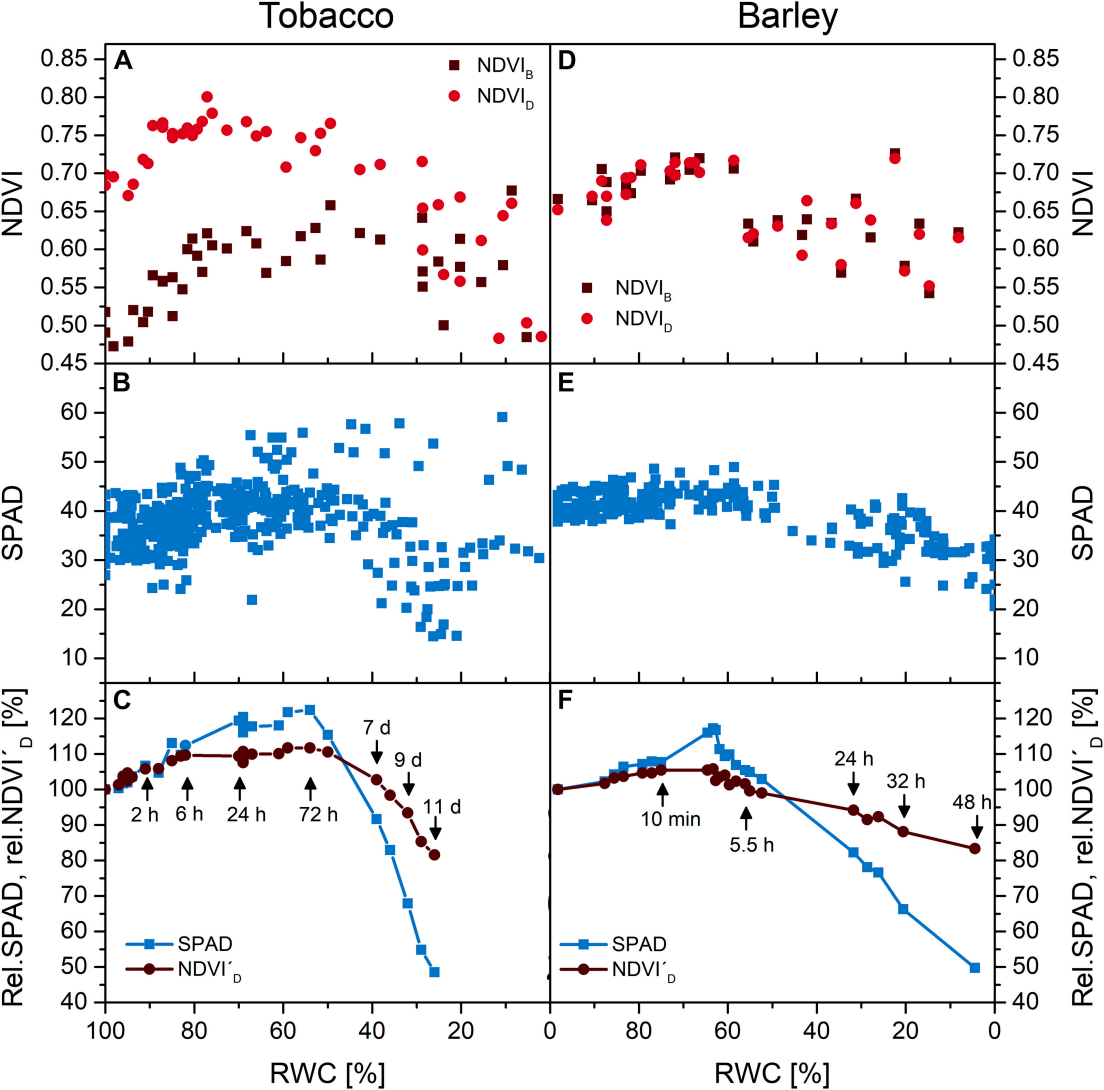
Normalized difference vegetation index [NDVI = (R_780_ − R_630_)/(R_780_ + R_630_)] estimated from measurement of diffusive R from the abaxial (NDVI_B_) and adaxial side (NDVI_D_) of desiccating leaves of tobacco (A) and barley (D). SPAD values of desiccating leaves of tobacco (B) and barley (E). Relative SPAD and NDVI′_D_ (estimated from measurement of directional R from the adaxial side) of a representative leaf of tobacco (C) and barley (F) during its desiccation (in % of the value measured immediately after leaf detachment). For selected data points, the time after the leaf detachment is indicated.

The leaf shrinkage was also reflected in NDVI and SPAD values (Fig. 5). Although the values were quite scattered, there was an obvious increasing trend in NDVI [from both abaxial (NDVI_B_) and adaxial (NDVI_D_) leaf side; Fig. 5A and D] and in SPAD (Fig. 5B and E) during the early stages of desiccation. This trend was more pronounced in tobacco, especially for NDVI_B_. As the leaves shrank, light absorption by Chl increased and both R and transmittance (at 630 and 650 nm, respectively) decreased, resulting in the increase of NDVI and SPAD. A subsequent decrease in NDVI and SPAD (Fig. 5) corresponded to a decrease in Chl content in leaves desiccating for a longer time (tens of hours).

The stated trends were more clearly documented by repeated measurements of NDVI and SPAD on selected leaves during their desiccation (Fig. 5C and F). In this case, we calculated NDVI′_D_ from the directional R_D_ measured with the PolyPen, as this measurement is much faster and gentler than the measurement of diffusive R used for the estimation of NDVI_B_ and NDVI_D_.

### Changes in Chl fluorescence parameters

**Fig. 6.**
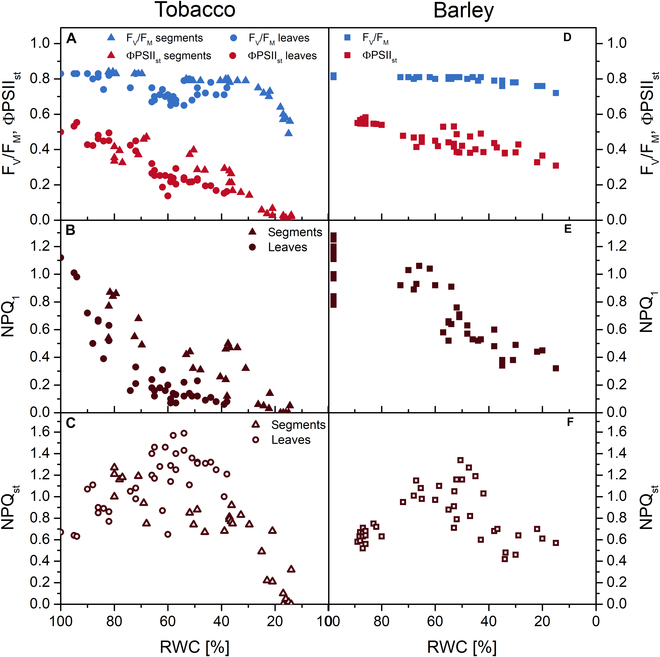
Chl fluorescence parameters of desiccating tobacco and barley leaf samples. (A and D) Maximum quantum yield of PSII photochemistry in the dark-adapted state (F_V_/F_M_) and effective quantum yield of PSII photochemistry in the light-adapted state (ΦPSII_st_). (B and E) Nonphotochemical quenching of Chl fluorescence after 1 min of exposure to actinic light (NPQ_1_). (C and F) Nonphotochemical quenching of Chl fluorescence at steady state (NPQ_st_). In case of ΦPSII_st_ and NPQ_st_, RWC was estimated from the average of the sample weight before and after the measurement. Each point represents a single leaf sample. For tobacco, in addition to slow desiccating whole leaves, circular leaf segments (14 mm diameter) were used to achieve a lower RWC in a comparable time as with barley leaves.

In addition to R measurements, in vivo Chl fluorescence can also be used to assess plant water status [2,22,27]. Therefore, selected Chl fluorescence parameters were measured in order to evaluate their sensitivity to the RWC decrease. A response of the maximum quantum yield of PSII photochemistry (F_V_/F_M_) to decreasing leaf RWC was very weak (Fig. 6A and D and Fig. S5), which is consistent with previously published results [23,27,39,44,45]. An effective quantum yield of PSII photochemistry in the (steady) light-adapted state of leaves, ΦPSII_st_, was much more sensitive in both tobacco and barley (Fig. 6A and D and Fig. S5).

Nonphotochemical quenching of Chl fluorescence (NPQ), both after 1 min of exposure to actinic light (NPQ_1_) and at steady state (NPQ_st_), was also quite sensitive. With decreasing RWC, NPQ_1_ decreased monotonically (Fig. 6B and E and Fig. S5), indicating a gradual slowdown of the light-induced activation of electron and proton transport and related nonphotochemical quenching processes [23]. In contrast, NPQ_st_ first increased and then decreased with decreasing RWC (Fig. 6C and F and Fig. S5). It indicates that in less desiccated leaves, there was a higher protective dissipation of excess energy (probably related to a greater inhibition of dark-phase photosynthetic processes compared to primary photochemistry), whereas in more desiccated leaves there was already damage to PSII function so that transport processes and protective nonphotochemical quenching were inhibited. These results confirmed our previous findings that ΦPSII and NPQ respond sensitively to the decrease in RWC in desiccating leaves of tobacco and barley [23,45]. The response of the Chl fluorescence parameters was mostly homogeneous across the leaf area (Fig. S5), reflecting a similar degree of impact on the photosynthetic function throughout the leaf.

In the case of Chl fluorescence parameters, not only whole tobacco leaves but also the prepared small circular segments were used for the measurement (Fig. 6 and Fig. S5), because whole tobacco leaves desiccated much more slowly than barley leaves (Fig. S1). Due to slow drying, in whole tobacco leaves, the effect of decreasing RWC could be combined with the effect of the onset of senescence, during which the photochemical function also deteriorates [46,47]. It is clear from Fig. 6A and B that the values of F_V_/F_M_, ΦPSII_st_, and NPQ_1_ were higher in the segments than in the leaves of similar RWC, which would correspond to the effect of senescence in whole leaves. In the case of NPQ_st_, this effect was not obvious (Fig. 6C), probably due to its biphasic change during the RWC decrease.

Interestingly, the response of the Chl fluorescence parameters of both leaves and leaf segments of tobacco was more sensitive than in barley (compare sections A to C with D to F within Fig. 6). This means that regardless of the desiccation rate and despite the slower decline in EWT, the photosynthetic function was more inhibited in the tobacco leaves.

### Comparison of the measured parameters based on the coefficients CR, CS, and CI

To compare the measured parameters in terms of their applicability for assessing leaf water status, we first estimated their *CR*. The parameters were then ranked from most to least reliable according to their *CR* values (Table and Figs. S6 and S7). Interestingly, the order of the parameters was quite different for tobacco and barley leaves (Table). In both species, the highest *CR* (and also *R*^2^) was found for ψ_psy_, which was expected due to the usually very close relationship between water potential and RWC (Fig. S8).

**Table.** Parameters measured on desiccating tobacco and barley leaves ranked according to the value of their coefficient of reliability (*CR*) within the RWC interval 100% to 50% and the corresponding values of the coefficient of determination (*R*^2^)

Ranking by *CR*	Tobacco			Barley		
	Parameter	*CR*	*R* ^2^	Parameter	*CR*	*R* ^2^
1	ψ_psy_	2.5024	0.8680	ψ_psy_	2.6967	0.8669
2	ψ_press_	1.2057	0.5869	WI_D_	1.5908	0.6585
3	NPQ_1_	1.2032	0.6746	ΦPSII_st_	1.4727	0.7203
4	ΦPSII_st_	1.1858	0.6309	WI_B_	1.0175	0.7968
5	NDVI_B_	1.0770	0.6449	WI_SWIR_	0.9821	0.5522
6	ΔR_B_	0.9035	0.5199	NPQ_st_	0.9550	0.5663
7	ΔR_D_	0.8307	0.4596	ψ_press_	0.5430	0.3059
8	SPAD	0.7382	0.2659	NPQ_1_	0.5177	0.4683
9	F_V_/F_M_	0.6686	0.4022	F_V_/F_M_	0.4441	0.3989
10	NPQ_st_	0.4870	0.2372	SPAD	0.4042	0.1768
11	NDVI_D_	0.4158	0.2044	ΔR_B_	0.2826	0.0832
12	WI_B_	0.1170	0.0026	ΔR_D_	0.2516	0.0867
13	WI_SWIR_	0.1066	0.0122	NDVI_B_	0.1975	0.0485
14	WI_D_	0.0018	0.000005	NDVI_D_	0.1397	0.0242

The coefficient of determination is defined as follows: $R^{2}=1-\frac{\sum_{i=1}^{n}{\left(y_{i}-y_{\mathrm{li}}\right)}^{2}}{\sum_{i=1}^{n}{\left(y_{i}-\bar{y}\right)}^{2}}$

In barley, the second highest *CR* was found for WI_D_. WI_B_ and WI_SWIR_ were ranked fourth and fifth, respectively. For tobacco, however, these parameters were ranked last (Table), which corresponds to their low sensitivity (Figs. 2 and 3). The lower sensitivity of WIs and WI_SWIR_ in tobacco might be expected to be associated with a longer maintained leaf water concentration (documented by the slower decline in EWT; Fig. 4) as a result of greater leaf shrinkage. The more pronounced shrinkage in tobacco was manifested by a higher *CR* for parameters reflecting leaf structure and Chl content, i.e., ΔR_B_, ΔR_D_, NDVI_B_, NDVI_D_, and SPAD. In barley, these 5 parameters had the lowest *CR* (Table).

The ranking of F_V_/F_M_ according to *CR* was the same for tobacco and barley (9th out of 14; Table). In tobacco, *CR* of ΦPSII_st_ and NPQ_1_ was relatively high due to their sensitive response to the RWC decrease, whereas *CR* of F_V_/F_M_ and NPQ_st_ was lower due to lower sensitivity (F_V_/F_M_) and biphasic dependence on RWC (NPQ_st_). In barley, F_V_/F_M_ had the lowest *CR* and, conversely, ΦPSII_st_ had the highest *CR* among the measured fluorescence parameters (Table).

In order to make a more detailed comparison of the applicability of the measured parameters, we estimated additional coefficients *CS* and *CI* and, similarly to the case of *CR*, ranked the parameters according to their sensitivity (based on the *CS* ranking) and inaccuracy (based on the *CI* ranking) (Tables S1 and S2). For tobacco, the ranking of the parameters according to *CS* largely corresponded to their ranking according to *CR*. Much poorer overlapping of parameters ranking by *CR* and *CS* was found in barley. However, when the dependence of *CR* on *CS* was plotted, a correlation was found not only for tobacco (*R*^2^ = 0.4522) but also for barley (*R*^2^ = 0.3559). On the contrary, no correlation was found between *CR* and *CI* (neither for barley nor for tobacco). Therefore, the reliability of a method was primarily determined by its sensitivity. A relative good correlation was found between *CS* and *CI* (*R*^2^ = 0.8173 for tobacco and *R*^2^ = 0.3912 for barley), which was anticipated because the more sensitive the method, the less accurate it is, as it reflects more the variability of the samples.

**Fig. 7.**
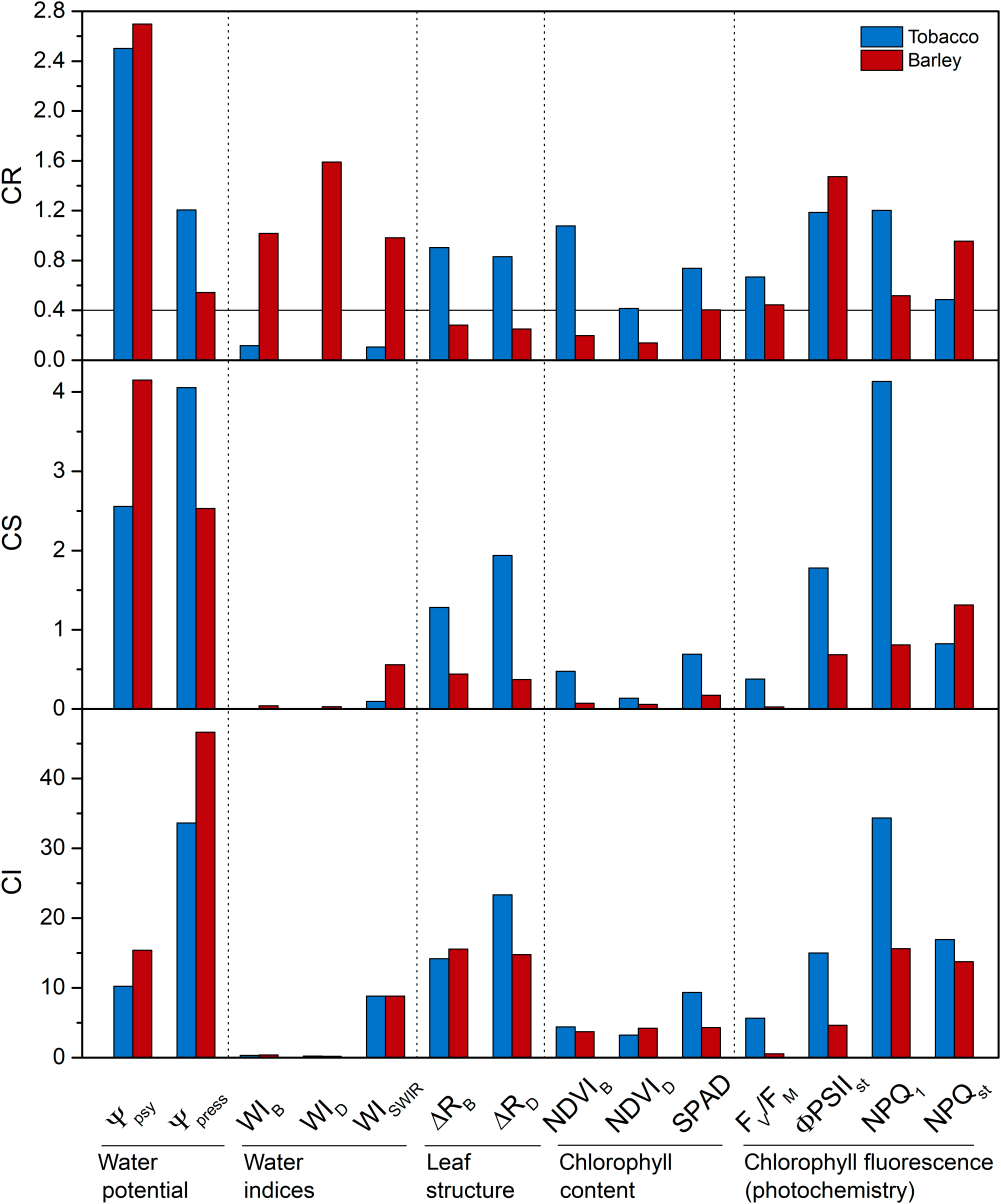
Coefficient of reliability (*CR*), coefficient of sensitivity (*CS*), and coefficient of inaccuracy (*CI*) of parameters measured in desiccating leaf samples of tobacco and barley within the RWC interval 100% to 50%. The parameters have been divided into 5 groups according to the type of leaf characteristics they reflect. A horizontal line in *CR* plot indicates the reliability threshold (*CR* = 0.4).

For the purpose of further analysis, we have divided the measured parameters into 5 groups reflecting different leaf characteristics: (a) leaf water potential (ψ_psy_, ψ_press_), (b) WIs (WI_B_, WI_D_, WI_SWIR_), (c) leaf structure (ΔR_B_, ΔR_D_), (d) Chl content (NDVI_B_, NDVI_D_, SPAD), and (e) Chl fluorescence (photochemistry) (F_V_/F_M_, ΦPSII_st_, NPQ_1_, NPQ_st_). In order to compare these parameter groups in tobacco and barley leaves, we summed up the ranking of the relevant parameters according to the individual coefficients (Table S3). The lower the sum of *CR*, *CS*, and *CI*, the more reliable, sensitive, or inaccurate the parameter group is. Figure 7 summarizes the *CR*, *CS*, and *CI* values of all parameters. Considering the range of *CR* values obtained, a value of 0.4 was chosen as the reliability threshold.

Figure 7 clearly shows that in the case of tobacco, in addition to water potential measurements, Chl fluorescence parameters, parameters reflecting leaf structure (ΔR_B_ and ΔR_D_), and parameters reflecting Chl content (in terms of its apparent increase due to leaf shrinkage) were applicable for assessing leaf water status. WIs including WI_SWIR_ were below the reliability threshold and seem to be the least suitable, mainly due to their very low sensitivity. The situation in barley was markedly different—here, it is the WI determinations that appear to be most suitable for indicating RWC decline (Table S3). Chl fluorescence was less suitable, and ΔR_B_, ΔR_D_, NDVI_B_, and NDVI_D_ were even below the reliability threshold (Fig. 7). Table S4 summarizes the time required and the destructiveness/nondestructiveness of the measurement as additional indicators of the applicability of the parameters used.

## Discussion

### Applicability of spectral R for estimation of leaf water status of desiccating tobacco and barley leaves

With the growing need to monitor the condition of plants in terms of their water supply, rapid, nondestructive, and remotely measurable parameters reflecting the water status of leaves are being sought. Many authors recommend the measurement of spectral R and R-derived WIs for this purpose. However, plant leaves are optically very complex and variable structures, so it is very difficult to predict if and how their optical properties (including R) will change during desiccation, and it seems impossible to draw a general conclusion about the applicability of spectral R for estimating leaf water status. Accordingly, published results are controversial regarding the sensitivity of spectral R, with both high and (very) low sensitivity to decreases in leaf water content or concentration reported.

The high sensitivity of R (whether diffusive or directional) to the decrease in RWC in leaves has been reported in a number of papers, both during slow desiccation due to insufficient water supply [12,42] and during rapid desiccation of detached leaves [6]. In general, a relatively pronounced increase in R has been observed in desiccating leaves not only at wavelengths where water absorbs but also for total R in VIS, NIR, and SWIR [6,9,42].

However, in our desiccating tobacco leaves, R in VIS and NIR was basically insensitive to the decrease in RWC, its increase being observed only in almost dry leaves (Fig. 2A and B). In the green region (around 550 nm), R actually decreased in leaves with RWC around 50% (together with transmittance; data not shown), most likely due to the leaf shrinkage, which increased leaf absorption in this region. Although Chl absorbs relatively weakly in the green region, leaves absorb a significant fraction of green light. Compared to blue and red light, which is strongly absorbed by Chl, green light penetrates deeper into the leaf (e.g., [48]), so if the leaf shrinks during desiccation, the absorbance (and absorptance) will increase and R will decrease more in the green region than in the blue and red regions. Similar decrease in R (at 540 nm) was found by Woolley [10] in desiccating cotton leaves when RWC dropped to 70%. A decrease in R in the VIS region during leaf desiccation was also mentioned by Thomas et al. [34], who associated this decrease with the leaves becoming “darker green”. Accordingly, the Chl content in our tobacco leaves appeared to increase during the RWC decrease to 50% due to their shrinkage, as indicated by the increasing NDVI and SPAD values (Fig. 5A to C).

In the tobacco leaves, the minimal changes in R in the NIR and SWIR region caused neither WI_B_, WI_D_, nor WI_SWIR_ to decrease until RWC dropped to about 50% (Figs. 2C and 3A and Fig. S6L to N). The WIs had the lowest observed *CR* and *CS* (Fig. 7), making their measurement the least suitable for estimating the water status of tobacco leaves within the methods used. In the case of barley, the sensitivity of the WIs was higher, as they started to decrease at RWC around 60% (WI_B_, WI_D_) or 80% (WI_SWIR_) (Figs. 2G and 3B). Their *CRs* were among the highest (Fig. 7), so the application of R-derived WIs in the case of barley is encouraged.

In some cases, the sensitivity of the R parameters increased when EWT was used instead of RWC [14,21], indicating that it is the water concentration and not the water content that primarily determines the changes in R [43]. However, while EWT may correlate with WIs better than RWC, it reflects rather leaf or plant tolerance to water loss than their water status. If the EWT is stable during water deficit, the situation regarding the water concentration in the leaves remains satisfactory, but it is not possible to know that the plant or the leaves have less water than they need. From this point of view, RWC is therefore a more appropriate metric than EWT [5].

In our case, EWT decreased more slowly in desiccating tobacco leaves compared to barley when RWC decreased to 50% (Fig. 4), which corresponds to the less sensitive response of tobacco WIs. However, the slower EWT decrease in the tobacco leaves is not sufficient to explain the R insensitivity, since the R spectra are also strongly influenced by an internal leaf structure [7,14,15]. We assume that the high sensitivity of R and R-derived parameters to RWC or EWT obtained in some studies was not related to the direct effect of water, but rather to its indirect effect on the internal structure of the leaves. This would be indicated by the reported increase in R over the whole range studied (mostly from 400 to 2,500 nm) as the leaves desiccated [6,42]. This explanation corresponds to the models of Ceccato et al. [14,15], who introduced the so-called “internal structure parameter” and showed that this parameter had a more pronounced effect on R than a decrease in EWT. According to Ceccato et al. [15], R in VIS is primarily affected by the Chl content and the internal structure parameter, in NIR by the internal structure parameter and the dry matter content, and only in SWIR by the effect of EWT together with the internal structure parameter and the dry matter content.

The major “structural cause” of the overall increase in spectral R is most likely an increase in the number of air–water interfaces due to water loss from the intercellular spaces [10,16]. This explanation is consistent with studies showing the opposite effect of leaf infiltration with water, i.e., a general decrease in R in VIS, NIR, and SWIR [8,49,50]. As summarized by Knipling [8], it is not the volume of airspace that is critical for R, but rather the number or total area of air–wall interfaces [8]. However, the increase in the total area of air–wall interfaces does not always occur during leaf desiccation [16,51], which could explain cases where spectral R was insensitive to changing leaf water status [7,16,34].

### Applicability of Chl fluorescence for estimation of leaf water status of desiccating tobacco and barley leaves compared to spectral R and other parameters measured

Another method used for the high-throughput assessment of plant and leaf water status is in vivo Chl fluorescence [2,22,25], as it reflects photosynthetic function [26], which is dependent on leaf water status [28]. However, not all Chl fluorescence parameters are sensitive to leaf water status [11,27,28], and the degree of their sensitivity may vary between plant species [23,45]. When we compared the measured Chl fluorescence parameters with the parameters derived from the spectral R (including the WIs), surprisingly the order of the parameters according to *CR* was diametrically different for the desiccating tobacco and barley leaves (if ψ_psy_ is not taken into account) (Table and Figs. S6 and S7). In tobacco, the Chl fluorescence parameters had the highest average *CR* (0.89). They were followed by parameters reflecting the leaf structure (ΔR_B_, ΔR_D_; average *CR* = 0.87) and Chl content (NDVI_B_, NDVI_D_, SPAD; average *CR* = 0.74). As mentioned above, the WIs (WI_B_, WI_D_, WI_SWIR_) had the lowest *CR* (average *CR* = 0.1). Conversely, for barley, the WIs had the highest average *CR* (1.2). They were followed by the Chl fluorescence parameters (average *CR* = 0.84) and parameters reflecting Chl content (average CR = 0.74). The lowest average *CR* (0.26) was found for ΔR_B_ and ΔR_D_.

These results showed that the applicability of individual methods for assessing leaf water status can vary considerably from case to case, apparently depending on the scenario of structural changes occurring during leaf desiccation. In tobacco, leaf shrinkage was greater (Fig. S4A), which on the one hand caused the greater sensitivity of the parameters reflecting leaf structure and (apparent) Chl content (NDVI, SPAD) (Fig. 5), but on the other hand contributed to the slower decline in EWT (Fig. 4) and the reduced sensitivity of the R-derived WIs (Figs. 2 and 3). The relatively high sensitivity of NDVI and SPAD to the rapid decrease in RWC could be used to assess the extent of leaf shrinkage, reflecting the ability of the leaf to maintain water concentration during desiccation. Consistent with this, a marked increase in SPAD during leaf desiccation has been reported in the drought-tolerant *Lantana camara*, whereas no increase was observed in the drought-sensitive *Malosma laurina* [39]. Since there was no increase in spectral R in desiccating tobacco leaves, we assume that the pronounced change in leaf structure was not associated with an increase in the total area of air–wall interfaces.

The sensitive response of Chl fluorescence parameters (Fig. 6 and Fig. S5) suggests that tobacco mesophyll cells lost water, resulting in inhibition of photochemical processes. This explanation may seem inconsistent with the slower decline in EWT in tobacco, but it is important to note that water can be lost from different parts of the leaf at different rates, depending on a number of extrinsic and intrinsic factors, including leaf and mesophyll structure [52,53]. The extent of shrinkage of different leaf compartments can be the same [44], but also different [54]. We assume that in our tobacco leaves, the rate of shrinkage during desiccation was probably similar in cells and intercellular spaces, which could also be the reason for the nonincreasing area of air–wall interfaces and therefore R insensitivity.

In the case of barley leaves, there was less shrinkage and a more rapid decrease in EWT during desiccation (Fig. 4 and Fig. S4B), as indicated by the higher sensitivity of the WIs and the lower sensitivity of the parameters reflecting leaf structure and apparent Chl content (Table and Fig. S7). The more rigid structure of barley leaves with parallel veins may explain their lower shrinkage. The smaller changes in Chl fluorescence parameters compared to tobacco indicate a lower effect of leaf desiccation on mesophyll cells.

The use of simple data analysis presented, together with the proposed coefficients reflecting not only the sensitivity of the parameters but also the inaccuracy of the methods, can help to decide whether to measure spectral R or Chl fluorescence to assess leaf water status in a particular case. Of course, if there is a need to compare the applicability of other methods (e.g., thermal infrared sensing), these can also be included in the analysis. In our case, Chl fluorescence was more reliable and universal for estimation of leaf water status than spectral R, but in other cases, spectral R may be more suitable.

### Chl fluorescence and spectral R in the context of remote sensing

When comparing the applicability of Chl fluorescence and spectral R measurements for remote sensing, a number of issues need to be considered. An important fundamental limitation of Chl fluorescence is its relatively low specificity, as stress-induced changes in Chl fluorescence parameters may be the same or similar for different stresses. In contrast to model experiments under controlled conditions, this can be a significant problem in field experiments, where the action of different stressors, usually in combination, is typical. Compared to Chl fluorescence, R in NIR and SWIR is more specific to changes in leaf water status.

The Chl fluorescence measurement we used also has significant technical limitations. These include the need to darken the samples prior to measurement and the limited mobility of the equipment, which would make measurement under field conditions technically quite complicated. A partial solution to these problems in ground-based measurements is the use of portable fluorometers and dark adaptation in leaf clips (e.g., [55]). However, such measurements are still not very effective from the perspective of field or canopy remote sensing. A more promising alternative for field conditions is a light-induced fluorescence transient (LIFT) method [56], which allows Chl fluorescence to be induced from a distance using subsaturating (actinic) measuring flashlets in fast repetition rate. This method offers noninvasive, high-throughput measurements of PSII efficiency under incident sunlight. LIFT parameters (e.g., Fq’/Fm’, an operating efficiency of PSII) were found to correlate closely with parameters of conventional Chl fluorescence methods including F_V_/F_M_ and NPQ [56]. The LIFT method has been successfully used in high-throughput field phenotyping of durum wheat under drought [3,33].

Another Chl fluorescence-based method with more promising potential for remote sensing is SIF, which is measured using field instruments (handheld or top of canopy) as well as airborne and satellite systems over large areas [57,58]. SIF measurements also do not require dark adaptation of samples, allowing for continuous real-time monitoring of plant stress responses, including the response to drought. However, although SIF parameters showed early and sensitive responses to plant water limitation in a number of studies [2,22,30,59,60], the interpretation of SIF changes is still debated, as they are significantly influenced by environmental conditions [2,33]. Furthermore, the SIF response to water deficit is a combination of physiological and nonphysiological effects [30,31], while also being associated with changes in canopy R [31,32]. It is becoming increasingly clear that correct interpretation of Chl fluorescence data is unlikely to be possible without information on canopy and leaf structure, which can be estimated from spectral R measurements [31–33].

In terms of use for remote sensing, spectral R is generally more suitable than Chl fluorescence because spectral imaging is more accessible than Chl fluorescence imaging and R traits are in many cases sensitive to changes in leaf water status [3,9,20,33]. It is currently clear that to utilize R effectively for this purpose, it is necessary to measure R hyperspectrally, i.e., including SWIR [11–15,20,21]. A certain problem in meeting this requirement is the relatively high price of hyperspectral instruments and, similar to fluorometers, the robustness of traditional hyperspectral spectrometers, which significantly limits their use in field conditions. However, because of technological advances, handheld and low-cost hyperspectral sensors are becoming available, allowing (among others things) in situ monitoring of leaf water status [5].

A specific drawback that complicates the use of spectral R to monitor water status is its dependence on the extent and type of leaf structural changes caused by water loss, as also indicated in this work. Structural changes during leaf water loss differ in different plant species [10,11,35,53], in plants and leaves of different ages [16], and also between the adaxial and abaxial sides of leaves [7,19,54]. The structural changes in desiccating leaves also depend on the conditions (light, temperature, water, and nutrient availability) to which the plants are exposed before and during the water shortage [51], as these conditions significantly influence the leaf structure and thus the optical properties of the leaves [18]. Furthermore, if the stress is acute (as in our case), the structural changes may be different from the slower desiccation of leaves attached to a plant stressed by water limitation. The relationship between various structural modifications of leaves during desiccation and their corresponding R responses warrants additional investigation.

## Conclusion

It is becoming increasingly clear that in order to accurately estimate leaf water status and monitor plant response to drought, it is desirable to combine as many plant parameters as possible and to take into account the influence of as many factors affecting plants and parameter measurements as possible. This is a very complex and complicated approach that cannot be done without extensive knowledge, sophisticated instrumentation and software for data processing, and radiometric, spectral, statistical, and other modeling [33,57,61]. In contrast, our comparative analysis is very simple to understand and implement, i.e., it is easier to use for plant physiologists, farmers, or breeders. Although measurements at the leaf level are a limitation for high-throughput phenotyping or remote sensing, they provide valuable information. Because of the comparison of different parameters reflecting different leaf characteristics, such measurements can help to reveal the scenario of desiccation-induced changes, which may differ depending on the plant species, the drought tolerance of the plant, and/or the duration and severity of the stress.

However, validation of our analysis is needed both for leaves attached to plants stressed by water limitation and for leaves of plants growing under field conditions, as changes in leaf structure may differ under different circumstances. For the same reason, validation of our methodology for other plant species is also desirable. The information obtained on the correlations between changes in structure, hyperspectral R, and Chl fluorescence parameters in leaves under changing water status can then be incorporated into remote sensing technologies and models to improve the prediction of plant stress response to drought, e.g., in relation to crop yield. This approach is fully consistent with a new promising trend of synergistically using information from multiple sensors or domains for plant (crop) stress detection, monitoring, and management [59,61].

## Data Availability

The data that support the findings of this study are openly available in Zenodo repository at https://doi.org/10.5281/zenodo.10997204.
