# Supplementary material for: What to Choose for Estimating Leaf Water Status—Spectral Reflectance or In vivo Chlorophyll Fluorescence?
Source: Plant Phenomics. 2024 Aug 29;6:0243. doi: 10.34133/plantphenomics.0243 (PMC11358408; doi:10.34133/plantphenomics.0243)
Supplement: Supplementary 1 — Figs. S1 to S8 Tables S1 to S4 [file plantphenomics.0243.f1.docx]

**Supporting information**

**Fig. S1** Decrease in relative water content (RWC) of leaf samples of tobacco and barley with time after their detachment

**Fig. S2** Micrographs of leaf structure of fresh tobacco (A) and barley (B) leaves.

**Fig. S3** WI_SWIR_ images of representative desiccating tobacco and barley leaf samples**.** Numbers above the samples indicate their RWC in %.

**Fig. S4** (A) Leaf area (in % of the area of fresh leaves) and (B) equivalent water thickness (EWT) during desiccation of tobacco and barley leaf samples.

**Fig. S5** Imaging of chlorophyll fluorescence parameters of representative desiccating tobacco and barley leaf samples. The maximum quantum yield of PSII photochemistry (F_V_/F_M_), the effective quantum yield of PSII photochemistry in the light-adapted state (ΦPSII_st_), the non-photochemical quenching of chlorophyll fluorescence after 1 min of exposure to actinic light (NPQ_1_), and the non-photochemical quenching of Chl fluorescence at steady state (NPQ_st_).

**Fig. S6** Dependencies of measured parameters on RWC (in interval 100-50%) in desiccating tobacco leaves and segments. The parameters are ranked from most to least reliable according to their coefficient of reliability (*CR*). All parameters are normalized to their mean value (*ȳ*).

**Fig. S7** Dependencies of measured parameters on RWC (in interval 100-50%) in desiccating barley leaves and segments. The parameters are ranked from most to least reliable according to their coefficient of reliability (*CR*). All parameters are normalized to their mean value (*ȳ*).

**Fig. S8** Leaf water potential measured by psychrometry (Ψ_psy_) and by pressure chamber (Ψ_press_) in desiccating leaves of tobacco (A) and barley (B).

**Table S1** Parameters measured on desiccating leaf samples ranked according to their coefficient of sensitivity (*CS*) within the RWC interval 100-50% in tobacco and barley.

**Table S2** Parameters measured on desiccating leaf samples ranked according to their coefficient of inaccuracy (*CI*) within the RWC interval 100-50% in tobacco and barley.

**Table S3** Ranking of measured parameters according to their coefficient of reliability (*CR*), sensitivity (*CS*) and inaccuracy (*CI*) in desiccating leaf samples of tobacco and barley within the RWC interval 100-50%. The parameters have been divided into 5 groups (the first column) according to the type of leaf characteristics they reflect.

**Table S4** Approximate time required for the measurement of the parameters used in the study and the destructiveness/non-destructiveness of the measurement.

**
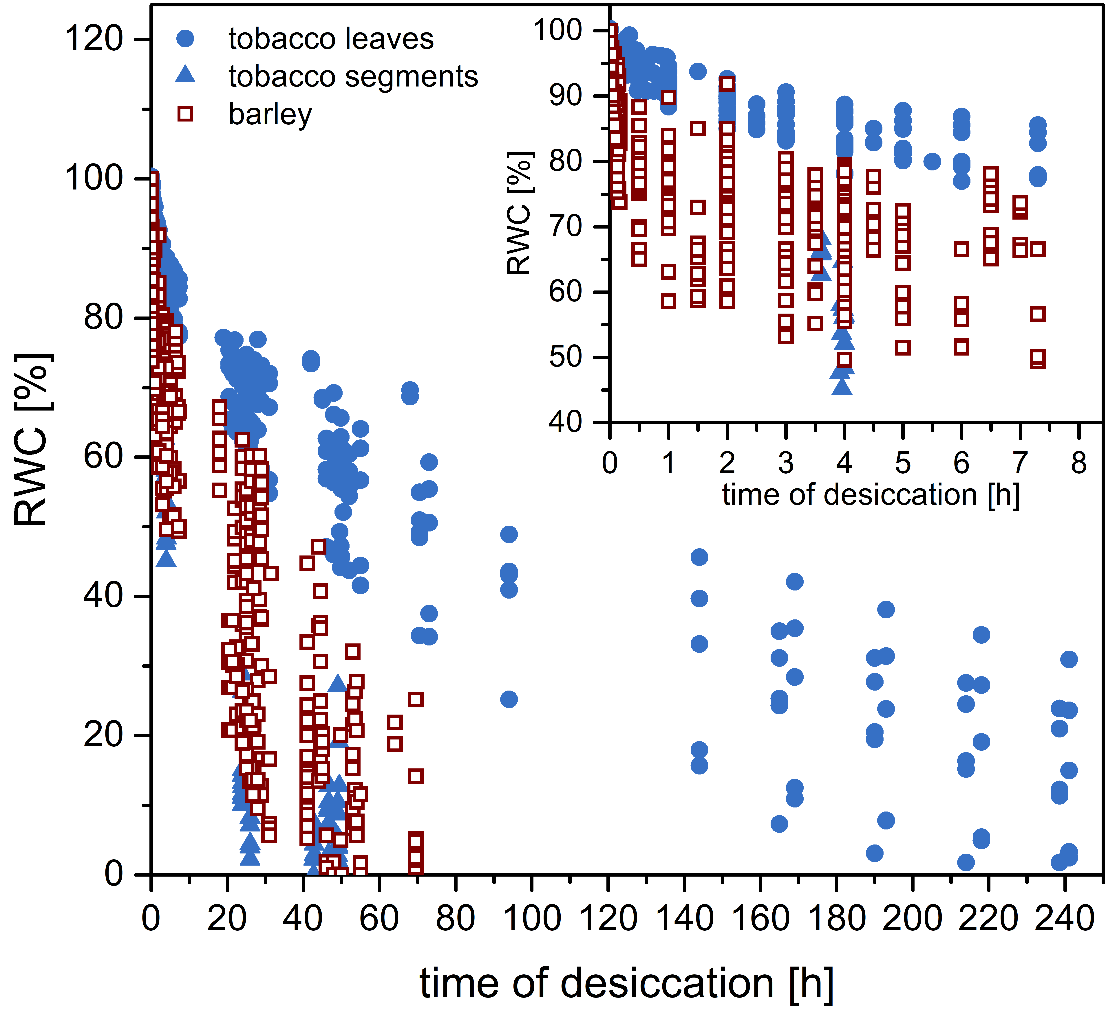
**

**Fig. S1** Decrease in relative water content (RWC) of leaf samples of tobacco and barley with time after their detachment

**
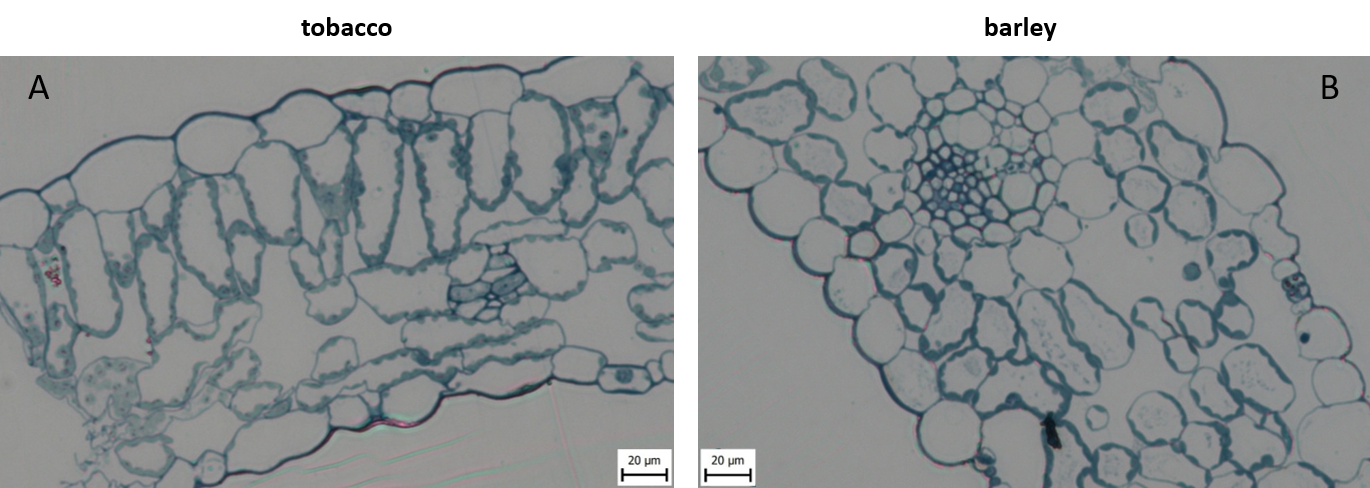
**

**Fig. S2** Micrographs of leaf structure of fresh tobacco (A) and barley (B) leaves.

**
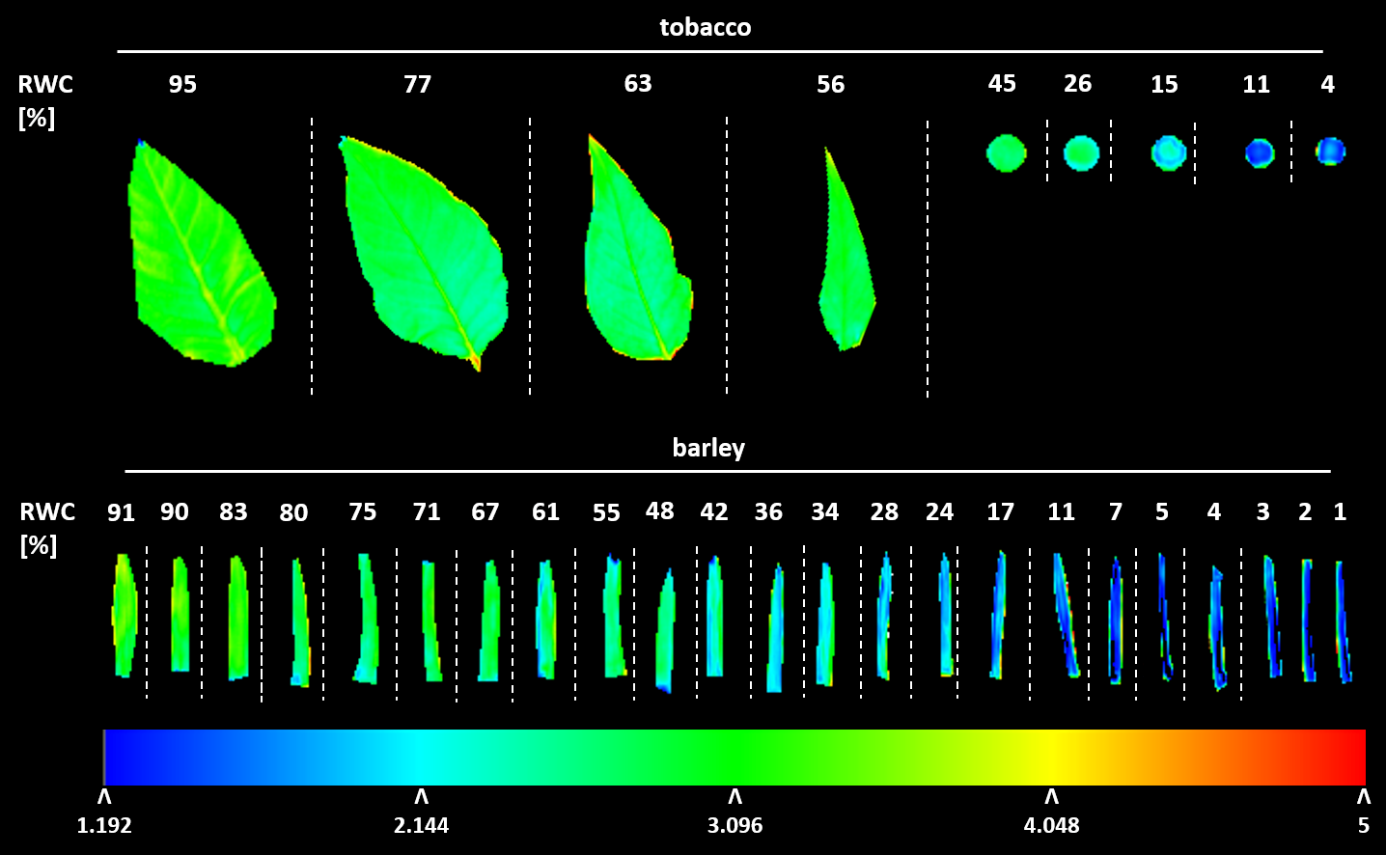
**

**Fig. S3** WI_SWIR_ images of representative desiccating tobacco and barley leaf samples**.** Numbers above the samples indicate their RWC in %.

**
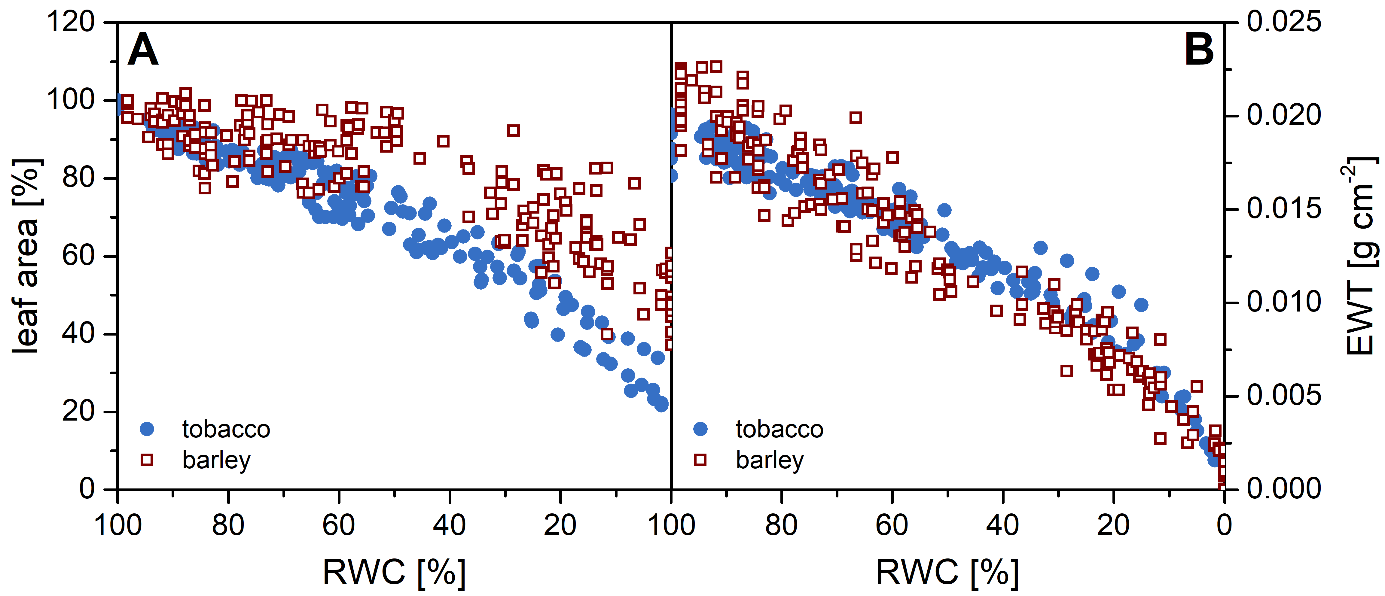
**

**Fig. S4** (A) Leaf area (in % of the area of fresh leaves) and (B) equivalent water thickness (EWT) during desiccation of tobacco and barley leaf samples.

**
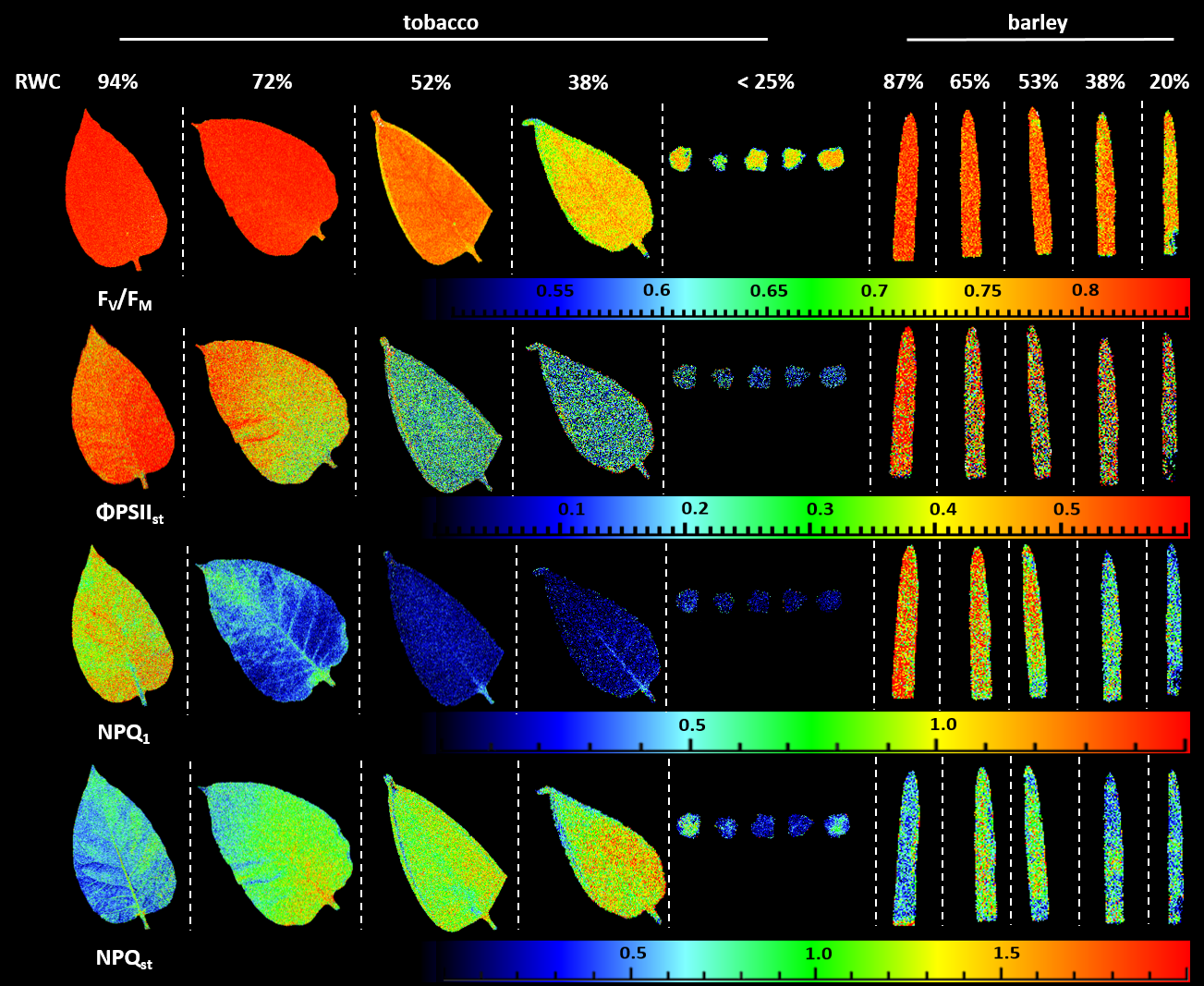
**

**Fig. S5** Imaging of chlorophyll fluorescence parameters of representative desiccating tobacco and barley leaf samples. The maximum quantum yield of PSII photochemistry (F_V_/F_M_), the effective quantum yield of PSII photochemistry in the light-adapted state (ΦPSII_st_), the non-photochemical quenching of chlorophyll fluorescence after 1 min of exposure to actinic light (NPQ_1_), and the non-photochemical quenching of Chl fluorescence at steady state (NPQ_st_).


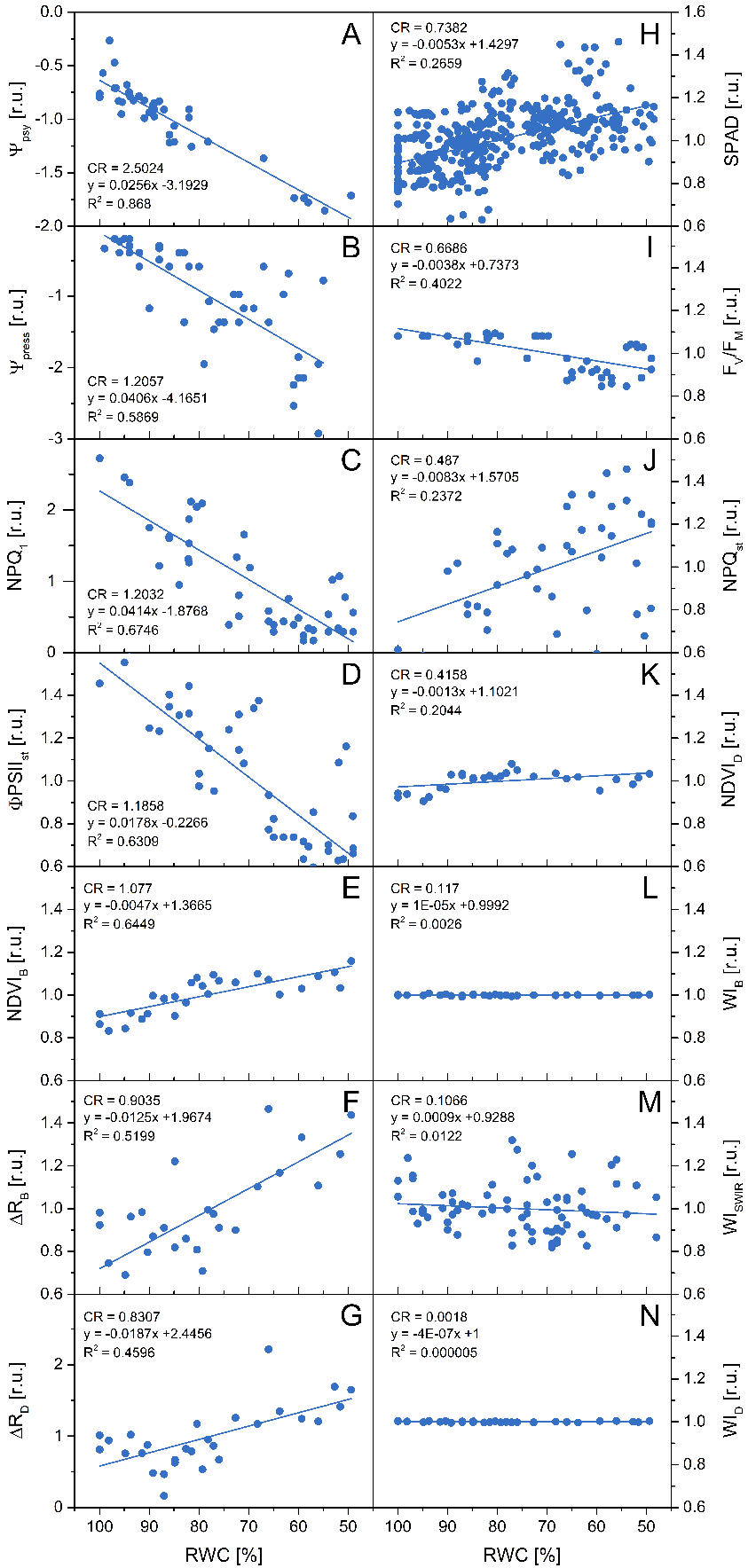


**Fig. S6** Dependencies of measured parameters on RWC (in interval 100-50%) in desiccating tobacco leaves and segments. The parameters are ranked from most to least reliable according to their coefficient of reliability (*CR*). All parameters are normalized to their mean value (*ȳ*).

**
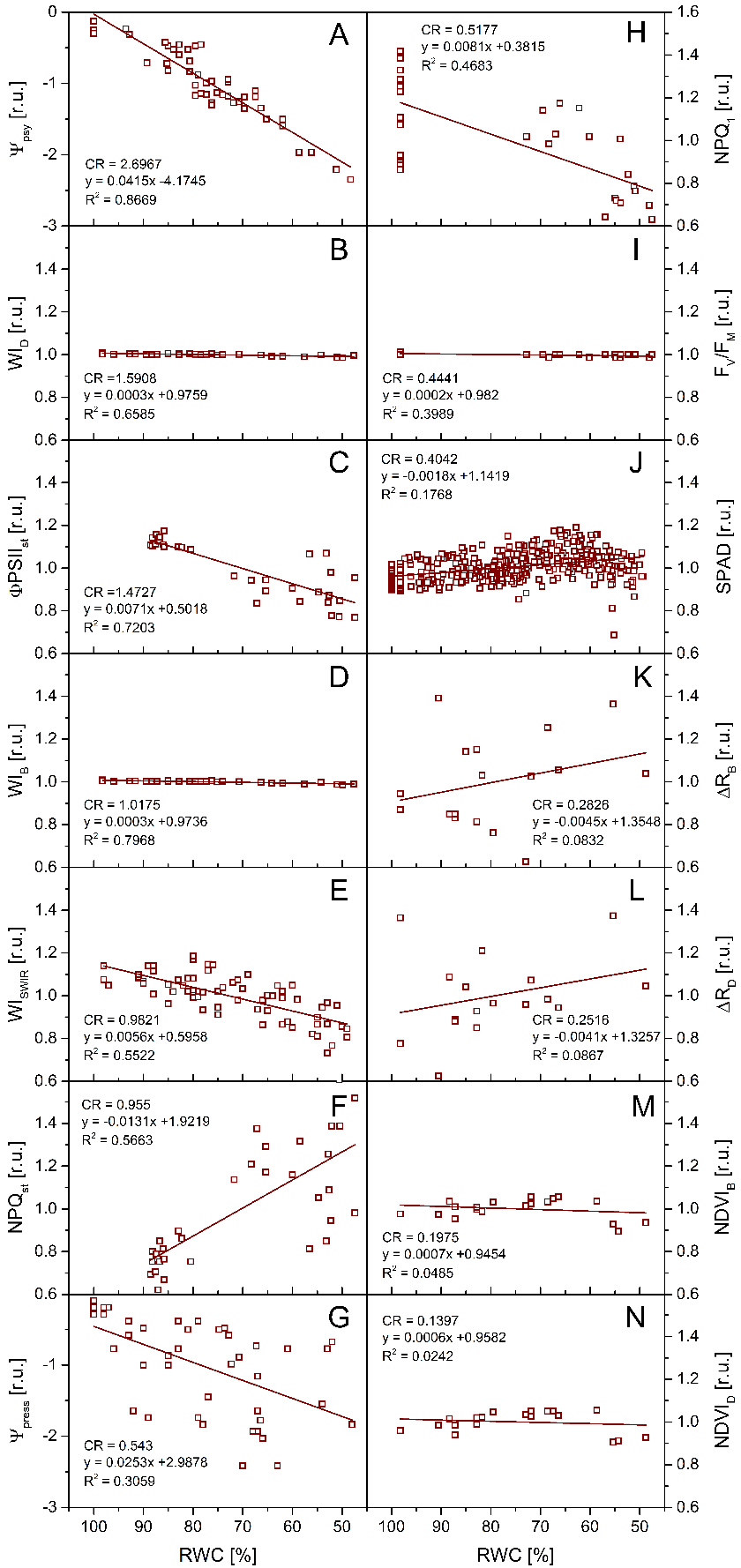
**

**Fig. S7** Dependencies of measured parameters on RWC (in interval 100-50%) in desiccating barley leaves and segments. The parameters are ranked from most to least reliable according to their coefficient of reliability (*CR*). All parameters are normalized to their mean value (*ȳ*).

**
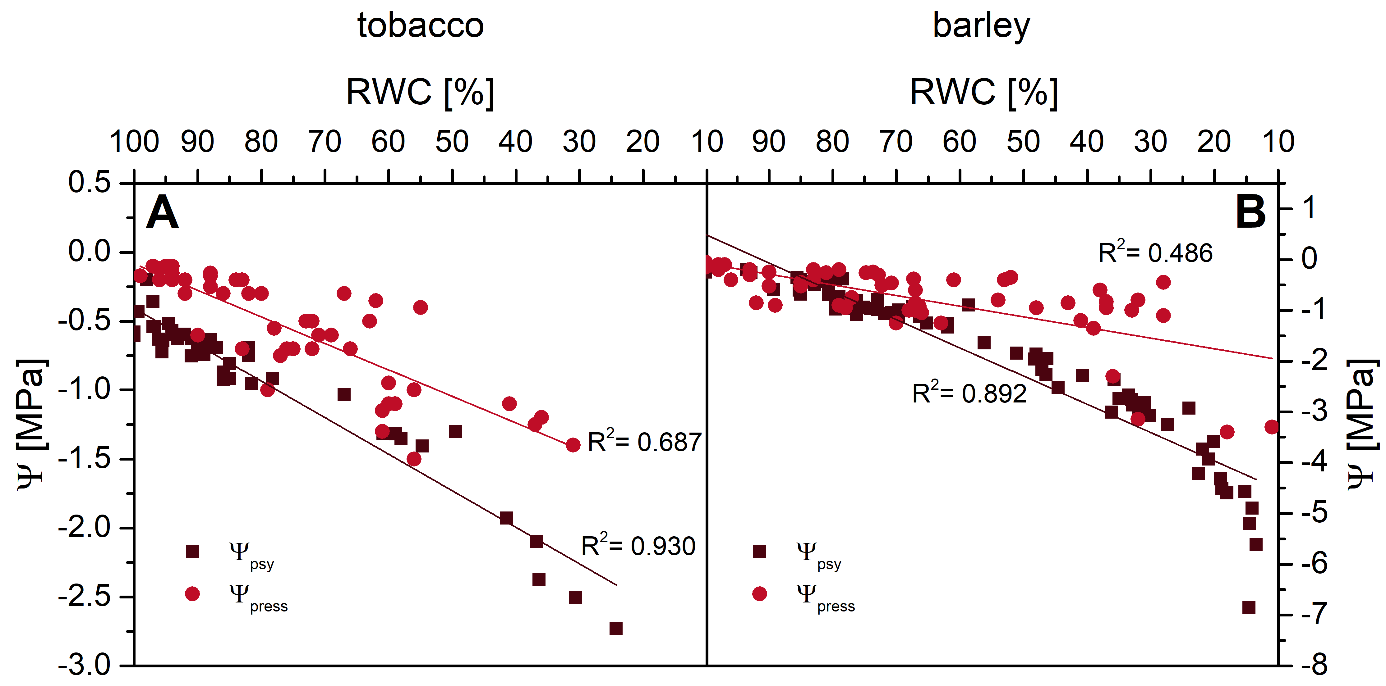
**

**Fig. S8** Leaf water potential measured by psychrometry (Ψ_psy_) and by pressure chamber (Ψ_press_) in desiccating leaves of tobacco (A) and barley (B).

|  | **Tobacco** | | **Barley** | |
| --- | --- | --- | --- | --- |
| **Ranking**  **by *CS*** | **parameter** | ***CS*** | **parameter** | ***CS*** |
| 1 | NPQ_1_ | 4.1322 | ψ_psy_ | 4.151 |
| 2 | ψ_press_ | 4.0544 | ψ_press_ | 2.532 |
| 3 | ψ_psy_ | 2.5562 | NPQ_st_ | 1.3143 |
| 4 | ΔR_D_ | 1.9394 | NPQ_1_ | 0.8084 |
| 5 | ΦPSII_st_ | 1.7802 | ΦPSII_st_ | 0.6856 |
| 6 | ΔR_B_ | 1.2813 | WI_SWIR_ | 0.5567 |
| 7 | NPQ_st_ | 0.8246 | ΔR_B_ | 0.4397 |
| 8 | SPAD | 0.6892 | ΔR_D_ | 0.3718 |
| 9 | NDVI_B_ | 0.4759 | SPAD | 0.1738 |
| 10 | F_V_/F_M_ | 0.3777 | NDVI_B_ | 0.0733 |
| 11 | NDVI_D_ | 0.1349 | NDVI_D_ | 0.0588 |
| 12 | WI_SWIR_ | 0.0941 | WI_B_ | 0.0391 |
| 13 | WI_B_ | 0.0039 | WI_D_ | 0.0293 |
| 14 | WI_D_ | 0.0001 | F_V_/F_M_ | 0.0247 |

**Table S1** Parameters measured on desiccating leaf samples ranked according to their coefficient of sensitivity (*CS*) within the RWC interval 100-50% in tobacco and barley.

|  | **Tobacco** | | **Barley** | |
| --- | --- | --- | --- | --- |
| **Ranking by *CI*** | **parameter** | ***CI*** | **parameter** | ***CI*** |
| 1 | WI_D_ | 0.2223 | WI_D_ | 0.1842 |
| 2 | WI_B_ | 0.3338 | WI_B_ | 0.3845 |
| 3 | NDVI_D_ | 3.2451 | F_V_/F_M_ | 0.5557 |
| 4 | NDVI_B_ | 4.4186 | NDVI_B_ | 3.7123 |
| 5 | F_V_/F_M_ | 5.648 | NDVI_D_ | 4.2113 |
| 6 | WI_SWIR_ | 8.8267 | SPAD | 4.301 |
| 7 | SPAD | 9.3367 | ΦPSII_st_ | 4.6556 |
| 8 | ψ_psy_ | 10.2149 | WI_SWIR_ | 8.8267 |
| 9 | ΔR_B_ | 14.1805 | NPQ_st_ | 13.7623 |
| 10 | ΦPSII_st_ | 15.0133 | ΔR_D_ | 14.7797 |
| 11 | NPQ_st_ | 16.9332 | ψ_psy_ | 15.3932 |
| 12 | ΔR_D_ | 23.3475 | ΔR_B_ | 15.5571 |
| 13 | ψ_press_ | 33.6285 | NPQ_1_ | 15.6153 |
| 14 | NPQ_1_ | 34.3444 | ψ_press_ | 46.628 |

**Table S2** Parameters measured on desiccating leaf samples ranked according to their coefficient of inaccuracy (*CI*) within the RWC interval 100-50% in tobacco and barley.

**Table S3** Ranking of measured parameters according to their coefficient of reliability (*CR*), sensitivity (*CS*) and inaccuracy (*CI*) in desiccating leaf samples of tobacco and barley within the RWC interval 100-50%. The parameters have been divided into 5 groups (the first column) according to the type of leaf characteristics they reflect.

|  |  | **Tobacco** | | |  | **Barley** | | |
| --- | --- | --- | --- | --- | --- | --- | --- | --- |
|  |  | **Ranking by** | | |  | **Ranking by** | | |
|  | **parameter** | ***CR*** | ***CS*** | ***CI*** |  | ***CR*** | ***CS*** | ***CI*** |
| Leaf water potential | ψ_psy_ | 1 | 3 | 8 |  | 1 | 1 | 11 |
|  | ψ_press_ | 2 | 2 | 13 |  | 10 | 2 | 14 |
|  | *Σ* | *3* | *5* | *21* |  | *11* | *3* | *25* |
|  |  |  |  |  |  |  |  |  |
| Water  indices | WI_B_ | 12 | 13 | 2 |  | 2 | 12 | 2 |
|  | WI_D_ | 14 | 14 | 1 |  | 4 | 13 | 1 |
|  | WI_SWIR_ | 13 | 12 | 6 |  | 5 | 6 | 8 |
|  | *Σ* | *39* | *39* | *9* |  | *11* | *31* | *11* |
|  |  |  |  |  |  |  |  |  |
| Leaf structure | ΔR_B_ | 6 | 6 | 9 |  | 11 | 7 | 12 |
|  | ΔR_D_ | 7 | 4 | 12 |  | 12 | 8 | 10 |
|  | *Σ* | *13* | *10* | *21* |  | *23* | *15* | *22* |
|  |  |  |  |  |  |  |  |  |
| Chlorophyll content | NDVI_B_ | 5 | 9 | 4 |  | 13 | 10 | 4 |
|  | NDVI_D_ | 11 | 11 | 3 |  | 14 | 11 | 5 |
|  | SPAD | 8 | 8 | 7 |  | 10 | 9 | 6 |
|  | *Σ* | *24* | *28* | *14* |  | *37* | *30* | *15* |
|  |  |  |  |  |  |  |  |  |
| Chlorophyll fluorescence (photochemistry) | F_V_/F_M_ | 9 | 10 | 5 |  | 9 | 14 | 3 |
|  | ΦPSII_st_ | 4 | 5 | 10 |  | 3 | 5 | 7 |
|  | NPQ_1_ | 3 | 1 | 14 |  | 8 | 4 | 13 |
|  | NPQ_st_ | 10 | 7 | 11 |  | 6 | 3 | 9 |
|  | *Σ* | *26* | *23* | *40* |  | *26* | *26* | *32* |

**Table S4** Approximate time required for the measurement of the parameters used in the study and the destructiveness/non-destructiveness of the measurement. The parameters that were used for the comparison according to their coefficients of reliability (*CR*), sensitivity (*CS*) and inaccuracy (*CI*) are written in bold.

| **Method** | **Instrument** | **Parameter(s)** | **Time requirement** | **Destructive** |
| --- | --- | --- | --- | --- |
| Spectral reflectance (diffusive) | spectroradiometer  with integrating sphere | R_B_, R_D_,  **ΔR_B_**, **ΔR_D_**,  **WI_B_**, **WI_D_**,  **NDVI_B_**, **NDVI_D_** | 15 min | yes |
| Spectral reflectance  (directional) | SWIR camera  PolyPen | **WI_SWIR_**  NDVI | minutes  seconds | no |
| Transmittance | chlorophyllmeter  SPAD-502 | **SPAD** | seconds | no |
| Chlorophyll fluorescence | fluorescence  imaging system | **F_V_/F_M_** | seconds | no |
|  |  | **NPQ_1_** | 1 min | no |
|  |  | **ΦPSII_st_, NPQ_st_** | 10 min | no |
| Psychrometry | psychrometric chamber | **ψ_psy_** | hours | yes |
| Pressure chamber | pressure chamber | **ψ_press_** | minutes | yes |
|  |  |  |  |  |
